# Chemical, Target, and Bioactive Properties of Allosteric Modulation

**DOI:** 10.1371/journal.pcbi.1003559

**Published:** 2014-04-03

**Authors:** Gerard J. P. van Westen, Anna Gaulton, John P. Overington

**Affiliations:** ChEMBL Group, European Molecular Biology Laboratory, European Bioinformatics Institute (EMBL-EBI), Hinxton, United Kingdom; UNC Charlotte, United States of America

## Abstract

Allosteric modulators are ligands for proteins that exert their effects via a different binding site than the natural (orthosteric) ligand site and hence form a conceptually distinct class of ligands for a target of interest. Here, the physicochemical and structural features of a large set of allosteric and non-allosteric ligands from the ChEMBL database of bioactive molecules are analyzed. In general allosteric modulators are relatively smaller, more lipophilic and more rigid compounds, though large differences exist between different targets and target classes. Furthermore, there are differences in the distribution of targets that bind these allosteric modulators. Allosteric modulators are over-represented in membrane receptors, ligand-gated ion channels and nuclear receptor targets, but are underrepresented in enzymes (primarily proteases and kinases). Moreover, allosteric modulators tend to bind to their targets with a slightly lower potency (5.96 log units versus 6.66 log units, p<0.01). However, this lower absolute affinity is compensated by their lower molecular weight and more lipophilic nature, leading to similar binding efficiency and surface efficiency indices. Subsequently a series of classifier models are trained, initially target class independent models followed by finer-grained target (architecture/functional class) based models using the target hierarchy of the ChEMBL database. Applications of these insights include the selection of likely allosteric modulators from existing compound collections, the design of novel chemical libraries biased towards allosteric regulators and the selection of targets potentially likely to yield allosteric modulators on screening. All data sets used in the paper are available for download.

## Author Summary

The physicochemistry and topography of ligand binding sites is generally conserved amongst related proteins, however, comparisons of the pharmacology of related targets (and even the same target) are often confounded by the existence of multiple, distinct, binding sites within the same protein. Importantly, these multiple binding sites can have ‘druggability’ or selectivity properties, and can therefore offer attractive novel approaches to develop new therapeutic agents. In this paper, sets of known ligands binding to the same target are classified as being either allosteric (binding at a site that is non-competitive for a natural ligand/substrate) or non-allosteric (binding at the same site as a natural substrate), it is demonstrated that there are differences in the profiles of ligands discovered empirically against these sites. Finally predictive models are developed with several useful applications in drug discovery.

## Introduction

### Allosteric modulators

The generation of drug-like lead and candidate molecules against a specific molecular target remains a major challenge in drug discovery. We are now in a position to partially understand the factors behind this, and they fall into two basic themes – 1) the diversity and size of the set of compounds used in the initial screen, and 2) the physicochemical properties of the binding site of the target, which may contain obligate features that are incompatible to binding molecules with drug-like properties [1]–[7]. There are now a large number of ‘tantalizing targets’, those that have strong biological rationale (for example genetic validation), but are currently outside the reach of the development of novel small molecule therapies. One strategy to avoid the issues of factor 2) above is to consider the development of allosteric regulators, which may have better, or at least differentiated physicochemical properties or advantages in selectivity and so forth [8]–[11].

The concept of allosterism has received ample attention in literature, yet the term is used relatively loosely, the current work starts by defining the definition of allosterism [8], [12]–[17]. Allosteric modulators are ligands for a biological target that exert their effect on this target via a mechanism that is not located at the molecular site of action of those ligands that are the natural ligands or substrates for this protein. Hence the term ‘allosteric modulator’ covers a very broad spectrum of compounds and it depends on the context and function of the protein in question what effect allosteric modulators truly have. Thus, while some papers have previously been published classifying allosteric modulators as a separate class of ligands in general, here it is argued that the physicochemical properties of the molecules depend equally on the target in question [11], [18].

For example if the target is a signaling protein (e.g. a G protein-coupled receptor (GPCR)) which naturally signals in response to ligand binding, an allosteric modulator can induce, inhibit, increase, or decrease this signal while still allowing the natural ligand to bind to the receptor (albeit with modified thermodynamic and kinetic parameters). In some cases the allosteric modulator can even prevent the natural ligand from binding through a conformational shift. Similarly, in the case of an enzyme, an allosteric modulator can increase, decrease, or block enzyme catalytic activity.

**Figure 1:**
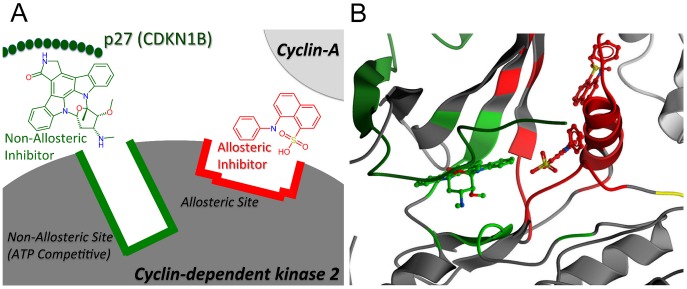
The concept of multiple binding sites on a single protein visualized schematically (A) and in protein data bank structure 1JSU (B). The ATP binding site was shown in green on cyclin dependent kinase 2 (CDK2) (grey), commonly referred to as the orthosteric binding site. One allosteric binding site (type V inhibitors) was shown in red, closely located to the orthosteric binding site. Also shown was a non-allosteric inhibitor (green, projected from PDB 1HCK) and an allosteric inhibitor (red, projected from PDB 3PY1). Finally cyclin-A was visualized pairing (light grey) with CDK2 and the natural inhibitor CDKN1B (dark green) to show the potential of allosteric inhibitors to disrupt the CDK2- cyclin-A protein-protein interaction.

In the case of proteins with multiple functions and active sites, categorizing ligands as allosteric versus orthosteric can be problematic. For example, Figure 1 shows cyclin-dependant kinase 2 (CDK2) involved in cell cycle control and known to have multiple binding sites for which multiple inhibitor types exist [19]. Firstly, several inhibitors are known to inhibit the protein *via* the ATP binding site (which is commonly referred to as orthosteric inhibition, type I inhibition). Hence *both* ligands in competition with the ATP-binding site *and* proteins in competition with the substrate to be phosphorylated could be deemed orthosteric but differ significantly in their physicochemical properties. However, in literature the latter group is also classified as allosteric inhibitors. Moreover, one naturally occurring inhibitor of CDK2, cyclin-dependent kinase inhibitor 1B or cyclin-dependent kinase inhibitor p27, binds to the complex of CDK2 – cyclin A and protrudes into the ATP binding site [20].

Conversely, several small molecule classes have been identified that inhibit protein kinases in an allosteric manner. Type II inhibitors occupy the nucleotide-binding pocket and extend into the allosteric pocket, stabilizing the enzymatic inactive conformation (DFG out), whereas type III inhibitors bind and occupy an allosteric pocket. Additionally, there are type IV inhibitors that are covalent inhibitors targeting reactive proximal cysteine residues [19]. Finally, a fifth class (Type V) of inhibitors have also been discovered for CDK2. These are non-ATP competitive, but the binding pocket has been shown to differ from that of known type II and III inhibitors [21]. The inhibitors have been shown to bind near the C-helix, which is involved in the interaction of CDK2 with cyclins A and E [22], [23]. Binding of these ligands also disrupts the protein – protein interaction (PPI) between CDK2 and cyclin, confirming the potential of allosteric modulators to disrupt PPIs.

Hence CDK2 is home to multiple binding sites to which multiple sets of ligands/substrates can bind in different ways. These ligands can be orthosteric (ATP-competitive), substrate competitive peptidomimetic molecules (non-competitive with regard to ATP, allosteric) or non ATP-competitive small molecules (allosteric), and can be further subdivided based on the mechanism of action. For these reasons, and because the approach here relies on retrieving allosteric papers, the term non-allosteric (rather than orthosteric) is used to describe other ligands binding to the same protein than those retrieved in the here described allosteric dataset. The definition of allosteric follows herein the target in question and general agreement in the literature; hence any observations are relative to this agreement in literature. There are a number of targets for which one can make similar distinctions with different forms of classification (e.g. in the kinase case one can define the ATP-competitive ligands as allosteric and only define the peptidomimetic ligands to be orthosteric). However, as the current results are derived from and based on medicinal chemistry literature it is chosen to follow this literature. Please see Case study 4 for further details on applying the here described methods to Kinase targets.

### Allosteric modulators as drugs

As discussed above, the differences in binding site properties relative to the substrate/agonist/antagonist site are potentially attractive for operational drug discovery reasons. Allosteric modulators can hit targets with natural ligands that are outside classic oral drug-like space (e.g. class B GPCRs), or are difficult to hit with specificity with regard to paralogs (e.g. class C GPRCs), or can even be used to distort protein-protein interactions [24]–[26]. In all of these cases allosteric modulators can allow modulation of these targets by small molecules using well-established medicinal chemistry and drug delivery strategies.

Furthermore, allosteric modulators are interesting from a physiological viewpoint, as they provide a way to modulate natural regulation (amplify a naturally regulated response) rather than completely inhibit or continuously activate proteins. Orthosteric drugs activate or inhibit a protein in a dose dependent manner. Yet allosteric drugs *can* differ, while their concentration in the body is dose dependent, their effect can be dictated only by concentration but can also be dictated by concentration in combination with physiological signaling and feedback loops [15].

Finally, in GPCR signaling allosteric modulators have been shown to possess other advantages over orthosteric ligands due to functional selectivity displayed by these allosteric ligands. Functional selectivity is expected to lead to greater selectivity and safety of drugs targeting GPCRs [27].

However, there are also less favorable characteristics of allosteric modulators making them less suitable as drugs. By definition allosteric modulators inhibit non-competitively and often via a secondary binding pocket. Hence the shape and pharmacophoric properties of such a pocket are not necessarily as highly conserved across paralogs and orthologs, as a catalytic/substrate site would be. The former site will usually not be under the same selective evolutionary pressure for protein function as the latter [28]. In the case of viral inhibitors or any other systems where rapid genetic mutation and selection is possible (e.g. anti-fungals, anti-bacterials and anti-cancer therapeutic areas), the use of allosteric modulators might lead to easier onset of resistance by point mutations. This is empirically the case of the non-nucleoside reverse transcriptase inhibitors (NNRTI) used in the treatment of infections with the Human Immunodeficiency Virus (HIV). NNRTIs are well known for a quick onset of (cross) resistance [29]. Moreover, they are only effective on the HIV-1 subtype and not on the closely related HIV-2 subtype (61% identical when comparing HIV-1 strain M with HIV-2 strain A). In HIV-2 the allosteric pocket cannot be formed due to the presence of substitutions native to HIV-2, which lead to NNRTI resistance in HIV-1. Conversely, non-allosteric inhibitors are effective on both strains due to their similarity to the natural ligands [29], [30].

### Improvement of bioactivity models

Public resources like ChEMBL [31], Pubchem [32], BindingDB [33], and Drugbank [34] have transformed many parts of drug discovery. The availability of the data enables new research into signaling processes and the ligand – target bioactivity space [35]–[37]. For example, computational models can be developed using existing compound structure and activity data, and used to predict potential activities for other compounds. Hence this data opens the door for new applications like *in silico* side effect prediction, personalized medicine and rational design of polypharmacological drugs [38]–[40]. However the presence of multiple binding sites and binding modes potentially confuses and frustrates model development and validation in cases where multiple binding sites exist. Consequently the ability to distinguish between mode of action and systematic characterization of these compounds could potentially prove invaluable in drug discovery.

### Aim of the work

In this work a top down analysis of allosteric modulators in the ChEMBL database was applied. Sets of ligands from papers in ChEMBL-14 were classified as being either allosteric, or non-allosteric (or presumed orthosteric) based on keywords, which were identified in both title and/or abstract. From the resulting papers the primary target was identified and then the compounds associated with this target were retrieved.

The resulting sets of ligands (allosteric and non-allosteric) are information dense (containing annotated target information, bioactivity, and the source documents). This information is subsequently exploited to study the allosteric concept over all bioactivities in ChEMBL, but also on a per target basis. Finally trends describing the chemistry, targets and bioactivity of compounds annotated to be allosteric are extracted

## Results/Discussion

### Composition of data sets

Allosterism has been reported in the ChEMBL database since the first indexed papers in 1980 (although the concept has been around in literature since the 1960's) [12], [13]. In total 987 unique documents were retrieved that together form the allosteric set (after manual curation for the case studies this number rises to 1,002). Likewise a non-allosteric set was retrieved, this set consisted of the documents that were not pulled in the first set and included the same restraints as applied to the allosteric set (see Methods). Finally a balanced non-allosteric set was derived from the full non-allosteric set to better perform unbiased classification. This balanced set was more similar in raw size and target distribution to the allosteric set (Table 1).

**Table 1:** Data set composition.

	Allosteric	Non-Allosteric (Balanced)	Non-Allosteric (Full)
Years	1980–2012	1980–2012	1980–2012
Documents	1,002	8,315	21,494
Data points	18,281	18,035	409,869
Assays	2,111	9,938	41,416
Binding assay derived	82%	81%	86%
Functional assay derived	17%	19%	13%
Other	1%	0%	1%
Targets	417	1,869	2,935
L1 target classes	10	13	13
L2 target classes	17	17	22
Compounds	17,829	17,709	384,288
Small Molecules	97%	96%	92%
Biologicals	3%	4%	8%
Organic (Small Molecule)	100%	99%	100%
Inorganic (Small Molecule)	0%	1%	0%
Peptide (Biological)	62%	46%	42%

Composition of the data sets generated. The allosteric set was obtained via text mining of abstracts; the non-allosteric (Full) set was the remainder of ChEMBL obtained using the same constraints as the allosteric set (e.g. limit bioactivity to primary assay). The non-allosteric (balanced) set was derived from the non-allosteric (full) set by taking a random percentage of each L2 target class present in the allosteric set. The classes ‘Organic’ and ‘Inorganic’ were subsets of the ‘Small molecules’ class. ‘Peptide’ was a subset of the ‘Biologicals’ class. Abbreviations: L1 – Level 1 target classification, L2 – Level 2 target classification.

**Figure 2:**
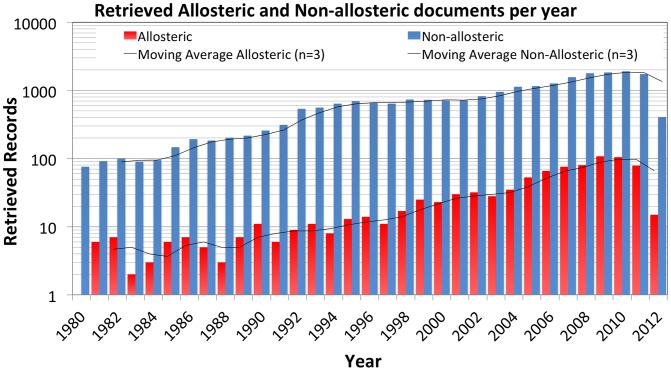
Distribution of retrieved allosteric and non-allosteric publications sorted per year. Overall the allosteric records made up a small fraction of the total records in ChEMBL-14. However a slight upward trend was seen. Note that the y-axis is logarithmic.

The allosteric records made up only a small fraction of the total records (around 3–4% of the total, Figure 2). However a trend was seen that the number of allosteric records have been increasing since the early 90's with a peak in 2009–2010. Possibly this increase was caused by the recent focus on allosteric modulation of GPCRs [14], [15], [17], [41], [42]. While the total number of allosteric records in 2012 was lower, this was likely caused by the fact that ChEMBL-14 does not contain an entire years' worth of 2012 publications. The full datasets are available for download on www.gjpvanwesten.nl/allosterism or ftp.ebi.ac.uk/pub/databases/chembl/Allosterism as are lists of all identified allosteric and non-allosteric activity_ids in ChEMBL-14.

### Target distribution

The next obvious question was: what targets are amenable to allosteric modulation? This information could be useful in assessing the likelihood of finding an allosteric modulator for related targets, and can also be input to screening or assay strategies. Ideally this information leads to insights how theses targets differ from the targets preferentially interacting with non-allosteric modulators. Since allosteric modulators are sometimes a secondary approach when non-allosteric modulation is infeasible or impossible, the expectation would be that the target distribution is different. Recently, Li *et al.* published work where they studied targets that can be allosterically modulated [43]. Yet, their work was limited to targets with known crystal structures, and hence would suffer from a systematic bias to simpler globular proteins. Here this was taken a step further investigating all allosterically modulated literature targets that are retrieved from ChEMBL. The targets in ChEMBL are classified within a hierarchy, in which level 1 (L1) denotes the protein type (e.g. ‘Membrane Receptor’ or ‘Enzyme’), L2 further narrows the protein family (e.g. Class A GPCR known as ‘7TM1’) and so forth down to individual proteins (supporting Figure S1) [31].

**Figure 3:**
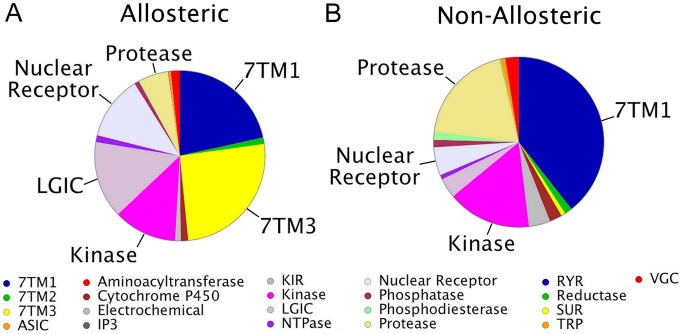
L2 target class distribution of both the allosteric (A) and non-allosteric data (B) sets. The distribution of the target classes differed between the two sets; which confirmed that targets that are easy to hit via non-allosteric inhibitors are not necessarily easy to hit via an allosteric modulator and vice versa. Abbreviations: 7TM1 - Class A GPCRs, 7TM2 - Class B GPCRs, 7TM3 - Class C GPCRs, IP3 - Inositol triphosphate receptors, KIR - Killer-cell Immunoglobulin-like Receptors, LGIC - Ligand Gated Ion Channels, RYR - Ryanodine Receptors, SUR - Sulfonylurea Receptors, TRP - Transient receptor potential channels, VGC - Voltage Gated Ion Channels.

Distinct differences were identified in the distribution of target classes when the total number of bioactivity measurements retrieved per target class was considered (L2 target distribution for both data sets, Figure 3). While the major target classes known from medicinal chemistry literature were represented in both sets (e.g. class A GPCRs and Proteases) their distribution differed between sets, moreover there were major differences [4]. For instance class C GPCRs were enriched among the allosteric set, as were the Nuclear Receptors and the Ligand-Gated Ion Channels. For class C GPCRs it has traditionally been difficult to obtain selectivity using non-allosteric ligands as these ligands tend to be very small [41]. The tight structure-activity relationships observed, centered around very ligand efficient recognition of the natural effector ligand do not allow much opportunity for variation in the receptor sequence and consequently in synthetic ligands biding this site (e.g. Metabotropic glutamate receptors, GABA_B_ receptors, etc.). However it has previously been shown that selectivity can be obtained using allosteric modulation and this course of action has been pursued in the literature and was hence represented in the data set [17], [41], [44]. The overrepresentation of GPCRs was expected as it has previously been shown that GPCRs are targets typically readily accessible to allosteric modulation [16], [42], [45]. A similar plot has been created for the L1 target class, which can be found in the supporting information (supporting Figure S2).

### Chemical structure properties

**Figure 4:**
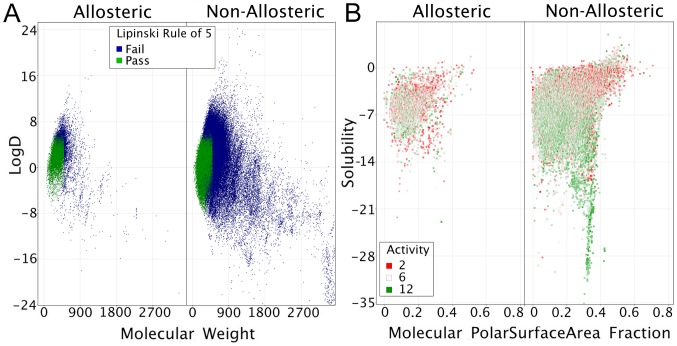
(A) Scatter plots showing the molecular weight (x), LogD (y) and adherence to the rule of 5 (color) of allosteric and non-allosteric compounds. The allosteric compounds represented a subset of the non-allosteric ligands; this image was conserved among most different target classes. (B) Scatter plots showing the molecular polar surface area fraction (x), solubility (y) and activity (color; pKi, pKd, pIC_50_, pEC_50_,). The area of high activity was observed to be narrower in the allosteric set versus than non-allosteric set. The non-allosteric compounds could display high affinity along a broader range of both properties.

Similar to the target-based overview of allosteric versus non-allosteric compounds, the chemical properties of both classes of compounds were investigated to highlight differences (Figure 4A). The two most important observations were that historically identified allosteric modulators tend to fall within a much more narrower range of molecular weight (but are a subset *of* non-allosteric compounds rather than distinctly separated *from* non-allosteric compounds) and secondly that allosteric modulators adhered slightly better to Lipinski's rule of 5 (75% versus 66%). Yet the important observation here was that the literature does not contain much information about allosteric modulators that are far from drug-like space. However, the relative scarcity of non drug-like allosteric modulators does not mean that these are not possible (e.g. the peptidomimetic kinase inhibitors). A similar observation has been made by Wang *et al.* yet some examples of allosteric modulators outside drug-like space were retrieved here, contrary to their work [11]. One possible explanation for this lack of non-drug-like allosteric modulators could be based on the bioactivity statistics of allosteric modulators (see below).

The differences between allosteric modulators and non-allosteric modulators were further explored in Figure 4B where normalized activity was also included (based on a negative log value of IC_50_, EC_50_, Ki and Kd values). Overlap was observed in the high affinity locations shared by allosteric and non-allosteric ligands in a scatter plot showing compound fractional polar surface area and molecular solubility. Yet non-allosteric compounds also showed high affinity at fractional polar surface and molecular solubility values outside the values preferred by the allosteric compounds. From these observations it was concluded that the allosteric modulators in literature form a more restricted range subset (in the sense of physicochemical properties) from the overall set of compounds.

Combined, these results demonstrate that allosteric compounds are not distinct from non-allosteric compounds, however, given historical data, they appear to form a subset of the broad non-allosteric compounds (or medicinal chemistry derived compounds). The results also showed that allosteric compounds on average had a larger similarity between *allosteric* sets binding different target classes than between *non-allosteric* compounds binding different target classes (when considering physicochemical properties). The differences were further demonstrated using a case study where the chemical differences are relatively large between the two sets.

### Case Study 1: Class B GPCRs

**Figure 5:**
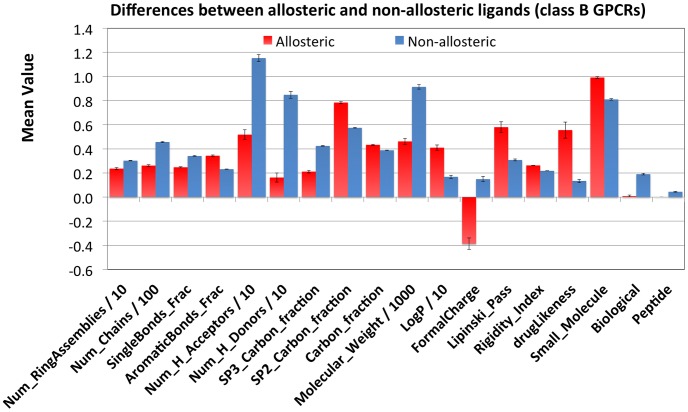
Mean value (and standard deviation) of several physicochemical properties calculated for both allosteric and non-allosteric ligands of Class B GPCRs. To plot all properties within one order of magnitude, a number of properties were scaled, dividing the mean value by 10 (e.g. logP) or by 1000 (e.g. molecular weight). Differences occurred for properties related to size (e.g. molecular weight, number of chains, number of hydrogen bond acceptors). However properties that were not correlated to size showed smaller differences (e.g. fraction of carbon). Note that the allosteric compounds were more rigid (higher sp2 hybridized carbon fraction, higher aromatic bonds fraction, higher rigidity index). For the full figure see supporting Figure S3.

As touched upon in the introduction, the desirability of allosteric modulators for a certain target is not only governed by physiological or pharmaceutical demands. There are cases where orthosteric modulation is not feasible for the development of orally active small molecule drugs. Example cases are the class B GPCRs for which the natural effectors are polypeptide ligands of typical length ranging 30 to 40 residues [25], [46]. There are many functionally and genetically validated links to pathology for this target class, and a number of drugs are available (some examples are iv/sc dosed - Calcitonin (Miacalcin), Exendin-4 (Exenatide), and PTH (Forteo)) [25], [47]–[49]. This target class was represented approximately equal in both the allosteric and non-allosteric data set (0.3% of the allosteric and 0.6% of the non-allosteric papers). While no large differences were apparent in the target distribution, the physicochemical properties of compounds annotated as allosteric modulators differed from those annotated as non-allosteric modulators. Figure 5 summarizes some of the findings for the class B GPCRs as retrieved from the data set. A figure with all 68 descriptors used (supporting Table S1) is also available (supporting Figure S3). In addition all data is available in tab delimited text format on www.gjpvanwesten.nl/allosterism or ftp.ebi.ac.uk/pub/databases/chembl/Allosterism. Here a limited figure is displayed for reasons of clarity.

Differences in physicochemical properties were found for allosteric and non-allosteric class B ligands (Figure 5). The non-allosteric (peptide like) ligands were very large (Mwt range 334 Da to 3591 Da for 95% of the data) whereas those ligands annotated to be allosteric modulators were ‘classical’ small molecules (Mwt between 305 Da and 569 Da for 95% of the data). Hence, differences were observed in properties related to size like: the number of chains or the number of hydrogen bond acceptors. However, when corrected for the size of the ligands, the differences were less distinct (e.g. carbon fraction of the total atoms). Interestingly the allosteric ligands were more rigid as indicated by a higher sp2 hybridized carbon fraction, lower sp3 hybridized carbon fraction, higher aromatic bonds fraction, and higher rigidity index (see methods for a further explanation of the rigidity index). Allosteric ligands tended to pass the Lipinski rule of five (60%) and were more drug-like, whereas non-allosteric ligands were less prone to pass Lipinski's rule (30%) and were not drug-like (Figure 5). Finally, the average formal charge for allosteric ligands was slightly negative and slightly positive for non-allosteric ligands. Similar charts have been created for all other significantly populated target classes (L2) and can be found on www.gjpvanwesten.nl/allosterism or ftp.ebi.ac.uk/pub/databases/chembl/Allosterism.

Secondary to physicochemical properties, substructures that are overrepresented in either the allosteric ligands or the non-allosteric ligands for a target class are of interest. Hence for each target class all present substructures (using circular fingerprints FCFP_6) were retrieved and their frequency in the allosteric and non-allosteric sets were compared against the background of the combined (full) set. Substructures were then sorted based on the enrichment score (supporting Table S2, Table S3, and Table S4). The results were in correspondence with what would be expected considering the natural ligands for these receptors and the observations from Figures 4 and 5. Substructures ranking high based on their allosteric score were quite specific, and tended to be aromatic. Conversely, substructures ranking very low based on their allosteric preference were small, frequently occurring and mainly introducing polarity. Interestingly, substructures scoring high based on their non-allosteric score included protein backbone like structures. The full set for all L2 target classes is available as a download from www.gjpvanwesten.nl/allosterism or ftp.ebi.ac.uk/pub/databases/chembl/Allosterism.

### Bioactivity of allosteric modulators

Protein targets and chemical properties of ligands in the allosteric set and the non-allosteric set were the point of focus in the above text. Now the differences between the bioactivity of allosteric compounds and the bioactivity of non-allosteric compounds are summarized. Considered were: potency (affinity), the number of targets that compounds from both groups have been tested on, the number of targets compounds from both groups were active on, the Ligand Efficiency (LE) [50], and a number of other efficiency indices (Binding Efficiency Index (BEI), Surface Efficiency Index (SEI), Normalized Surface Efficiency Index (NSEI), etc. [51], [52](Table 2)

**Table 2:** Bioactivity measurements for allosteric and non-allosteric compounds.

	Allosteric Median (MAD)	Non-Allosteric Median (MAD)
Activity	5.96 (±1.02)	6.66 (±1.17)
LE	0.319 (±0.0723)	0.310 (±0.0689)
BEI	16.3 (±3.69)	15.9 (±3.55)
SEI	11.1 (±4.12)	10.3 (±3.82)
NBEI	0.233 (±0.0528)	0.226 (±0.0503)
NSEI	1.16 (±0.370)	1.08 (±0.348)
*n*BEI	7.34 (±1.06)	8.12 (±1.19)
*m*BEI	8.49 (±1.06)	9.27 (±1.19)
Targets Annotated	2 (±1)	3 (±2)
Targets Active	0 (±0)	1 (±1)
Targets Inactive	1 (±1)	1 (±1)
Targets Other	1 (±1)	1 (±1)
Actives (%)	33	47
Inactives (%)	39	29
Other (%)	28	24

A threshold of 6 log units was used to classify compounds as ‘active’. Abbreviations: MAD – Median Average Deviation, LE – Ligand Efficiency (kcal/mol per non-hydrogen atom), NBEI – Normalized Binding Efficiency Index (non-hydrogen atoms), BEI – Binding Efficiency Index (molecular weight), SEI – Surface Efficiency Index (polar surface area/100), NSEI – Normalized Surface Efficiency Index (polar atoms), *n*BEI – Normalized Binding Efficiency Index taking the log after calculation of the ratio (non-hydrogen atoms), *m*BEI – Normalized Binding Efficiency Index taking the log after calculation of the ratio (molecular weight). See Abad-Zapatero *et al.* [52].

The median potency was lower for allosteric modulators than for non-allosteric modulators (5.96 log units versus 6.66 log units, p<0.01). Moreover, a lower fraction of the compounds was considered ‘active’ (33% versus 47%), with activity being defined operationally as potency better than micromolar (6 log units) or annotated ‘active’ in the source data. Likewise a higher fraction was inactive (39% versus 29%, less than 6 log units or annotated ‘inactive’ in the source data). Allosteric modulation is a process that cannot be explained by only ligand affinity (the dynamics are much more complicated and the reader is referred to a number of reviews) [53], [54], yet the current findings with regard to affinity are discussed here given the importance of this measurement in drug discovery.

Several possible explanations for the observed differences can be considered. Firstly, it is known that metabolites can be allosteric regulators and these metabolites can be present locally at very high concentrations and can hence exert their effect with a relatively low potency. Table 2 could implicate that the data set reflects the presence of metabolites in our dataset, annotated as allosteric ligands. High concentration metabolites would not need micromolar affinity when they are present at a millimolar concentration locally [8], [55], [56]. Secondly, another explanation can be that the optimization of high affinity allosteric binders is more challenging given the more constrained chemical characteristics that allosteric modulators display compared to non-allosteric modulators. However, there are two more likely but also more complex potential explanations for the observed lower affinity as will be described below.

### Rationalizing the observed lower affinity of allosteric modulators

A third explanation for the observed lower affinity could be derived from observations in the field of GPCRs. The current work is not the first to observe a lower affinity for allosteric modulators compared to non-allosteric interactions, in particular in the field of GPCRs this has been observed before [54]. While GPCRs are a complex modeling system given the baseline presence of both an orthosteric (natural ligand) and allosteric (G protein) binding site in all GPCRs, there are some observations that can perhaps be translated to a more general view of allosterism. It has been shown that allosteric interactions have a direct effect on the affinity of non-allosteric ligands (orthosteric in GPCRs) [54]. Given that affinity is defined as the ratio of ligand association to ligand disassociation rates, allosteric modulators directly affect the non-allosteric (dis)association rate. However, the allosteric interaction between two sites has been shown to be reciprocal [54], hence the affinity of allosteric modulators is influenced by the affinity of non-allosteric modulators. As such the observation of the lower affinity of allosteric modulators might be a product of the dominant usage of radio-ligand binding assays (as follows). Typically radio-ligand binding assays are set up using a well-known ligand, a radioactive molecule is synthesized based on this ligand and the binding of uncharacterized molecules is explored through their effect on the radio-ligand. Given that the radio-ligand is usually a well-known ligand, it is often a ligand with a reasonably high affinity. Hence this high affinity effect might influence the observed affinity of allosteric ligands due to the reciprocal nature between the binding site of an allosteric ligand and a non-allosteric ligand. When comparing competitive inhibition between two non-allosteric ligands (radio ligand and unknown molecule) this effect will likely not be present. While this explanation is funded on observations from the field of GPCRs, it should be noted that in this field allosteric modulation has arguable been the most intensely explored.

Another observation from the field of GPCRs is that ligand efficacy does not necessarily correlate to ligand affinity. There is documented evidence in literature wherein the ligand with the best affinity does not display the best efficacy [57], [58]. It has been hypothesized that this discrepancy can partially be explained through the concept of binding kinetics. For a number of GPCRs it has been found that efficacy is better explained when receptor residence time or disassociation rate is considered (the most efficacious ligands are shown to be the ligands with longer residence time) than when only affinity is considered [57]–[59]. In the case of allosteric modulators a similar principle might apply. Indeed, cases in which allosteric modulators modify binding kinetics of non-allosteric ligands have been described in literature [60], [61]. Given that we observe here that allosteric modulators tend to be relatively small and lipophilic molecules one can expect de-solvation to play a major role in binding kinetics. Hence these molecules might display a baseline longer residence time than non-allosteric molecules due to their physicochemical properties. However, further research and experimental evidence is required to confirm or reject this hypothesis.

### Implications for assays focused on allosteric modulation

While any classification into ‘active’ or ‘inactive’ is based on a cut-off, the observations here regarding affinity illustrate a larger issue. In screening efforts cut-offs are important to retrieve interesting ligands. If the median potency of allosteric modulators is lower than that of non-allosteric modulators (corroborated by the tendency of allosteric ligands to be smaller, to be more lipophilic, and to possess less hydrogen bonding potential) this could very well lead to possible allosteric modulators being missed in screening efforts. The general threshold for activity in primary screening is 10 µM to find compounds that are shown to have a median activity of 6.66 log units in ChEMBL. Hence, the implication would be that any screening effort for allosteric modulators (median activity of 5.96 log units) would need to be more sensitive or at least have the definition of ‘active’ adapted to conform to our observations. Moreover, given the reciprocal nature of the effects that allosteric and non-allosteric sites have on each other, it would be recommended to not use a single radio-ligand if one is aiming to find new allosteric modulators. A better choice is to use a spectrum of assays with different radio labeled ligands as has also been suggested by May *et al.* [54].

That said, allosteric compounds were found to have similar but slightly higher median binding efficiency indices (LE, BEI, SEI, NSEI), this difference was likely caused by the fact that allosteric modulators tend to be smaller than non-allosteric modulators. This potentially indicates on average smaller, less polar binding sites for allosteric versus non-allosteric classes [43]. Moreover, we observed that allosteric modulators tend to have been annotated to a lower number of targets (2 versus 3) but this difference is marginal. Additionally, the median number of targets a compound is active on is shown to be 1 (average 1.43) for the non-allosteric set, in line with the findings of Hu and Bajorath [62], but the values are median 0 (average 1.40) for the allosteric set.

In conclusion, allosteric modulators were found to be able to modulate targets with low affinity but high efficiency. In addition, the data did not show allosteric modulators to be inherently promiscuous binders – at least as inferable from the distribution of assays reported in ChEMBL –, rather there was a trend for allosteric compounds to be less promiscuous than non-allosteric modulators, which is also seen in previous work [43]. While the potential of the current data set is demonstrated by comparing the allosteric and non-allosteric set, this analysis is by no means exhaustive. Similar analyses can be performed comparing different allosteric sets or for instance comparing class C GPCR ligands from the allosteric set with the class A GPCRs of the non-allosteric set (comparing two different sets of trans-membrane domain binding ligands). Moreover, it should be noted that further research is required to determine if the lower binding affinity observed results from database bias or if this is an intrinsic property of allosteric modulators (and if so, what the cause is of this observation).

### Allosteric classification models

Above it was shown that there are chemical differences between allosteric ligands for a certain target class and non-allosteric ligands for that same target class. In some cases these differences were large (as in the case of class B GPCRs) whereas in other cases the differences appeared to be smaller (as in the case of class A GPCRs). These chemical distinctions were used to train a classification model that would be able to predict if a compound would likely be an allosteric modulator or a non-allosteric modulator for a given target based on the physicochemical properties. These models were created on the balanced set to avoid a large bias in classifier predictions (Table 1). Non-balanced models have also been trained and data is available in the supplementary information.

The use of (circular) fingerprints in the full (non-target specific) models was sidestepped for several reasons; firstly these models should have a large applicability domain and should hence not be limited to certain chemical motifs. Secondly, (chemical) sampling bias of specific historical target classes was to be avoided. Thirdly, the large chemical diversity would probably make those features that are predictive very generic (as shown in the class B GPCR case study for substructures negatively associated with allosteric modulators, supporting Table S2, S3, S4). Finally the improvement of circular fingerprints to the models was marginal (on average 5% as calculated by the average of the used parameters, supporting Table S5). Hence circular fingerprints were only used in more congeneric chemical sets (e.g. target specific) [63]. Models were judged by recall of allosteric modulators (Sensitivity (sens)); recall of non-allosteric modulators (Specificity (spec)); precision for allosteric modulators (Positive predictive value (PPV)); precision for non-allosteric modulators (Negative predictive value (NPV)); and Matthews correlation coefficient (MCC). These were all 0 for a non-predictive/random model and 1 for an ideal model with the MCC also potentially being -1 for an ideal inverse model (see Methods for further details).

**Figure 6:**
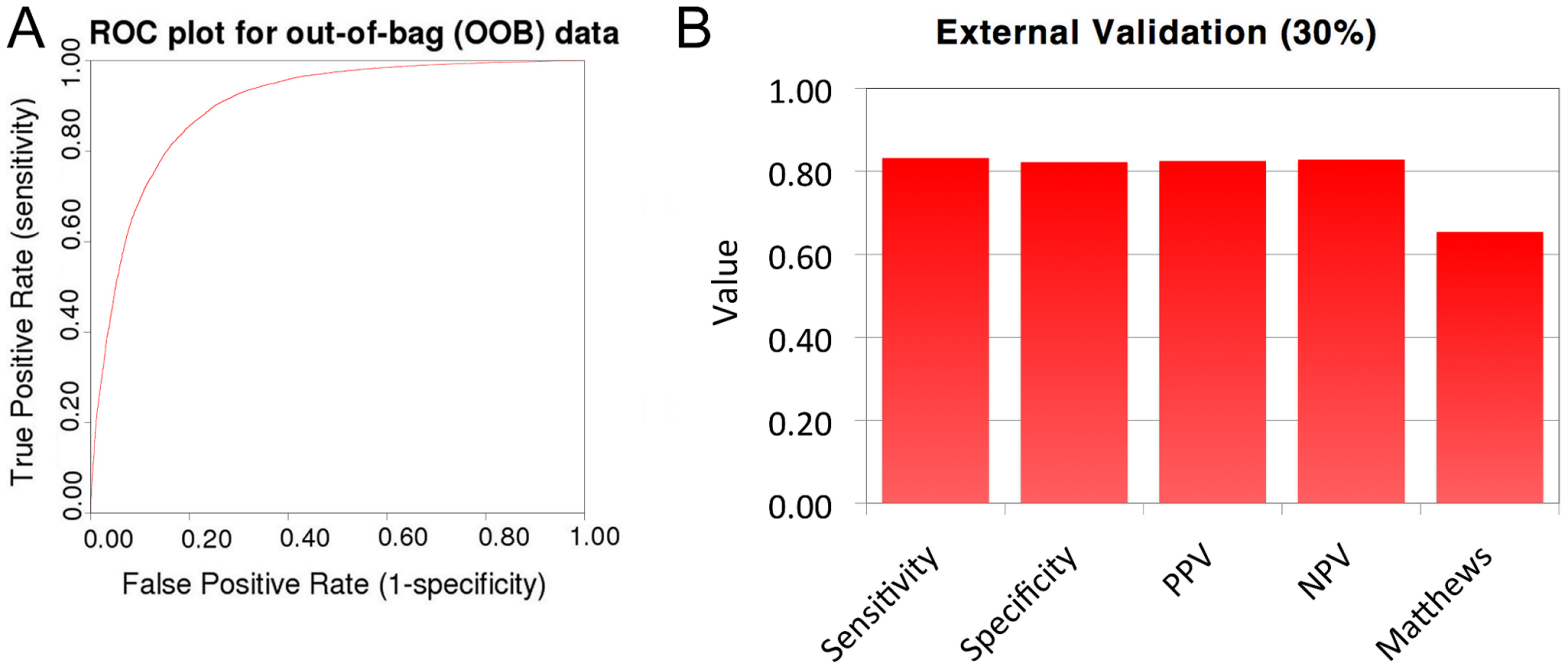
(A) Receiver Operator Characteristics (ROC) curve for out-of-bag validation of the allosteric classifier trained on 70% of the allosteric and balanced orthosteric set demonstrated good performance. (B) External validation on the remaining 30% of the data set confirmed good predictive performance.

Table 3 shows a selection of the results for allosteric classification models (each trained on 70% of the data and externally validated on the remaining 30%). For the full table see supporting Table S6, here we limited ourselves to a single page for reasons of clarity. Different models on data sets grouped by class L0 (protein binding compounds), L1 (first level classification), and L2 (second level classification) have been trained. Figure 6 shows the out-of-bag ROC curve and external validation for the L0 model. For all groups models were able to classify a compound as allosteric modulator or non-allosteric modulator of a given target class with good accuracy, yet model performance improved when sets became more specific (limited to a target class). These models provide a useful tool for the elucidation of the mechanism of action for compounds identified in primary HTS screening efforts.

**Table 3:** Examples of allosteric models for balanced data sets of L0, L1, and L2 groups.

Target Level	Class	Allosteric	Non-allosteric	Sens	Spec	PPV	NPV	Allosteric property 1	Allosteric property 2	Allosteric property 3	Non-allosteric Property 1	Non-allosteric Property 2	Non-allosteric Property 3
0	n/a	18,281	18,035	0.83	0.82	0.82	0.83	Doublebonds Frac	Carbon Frac	LogP	drugLikeness	PSA Frac	Stereoatom Frac
1	Enzyme	9,531	8,425	0.84	0.81	0.83	0.82	DoubleBonds Frac	Aromatic Bonds Frac	Sulphur Frac	drugLikeness	Molecular PSA	Num Chains
1	Ion Channel	1,974	1,966	0.86	0.88	0.88	0.86	LogD	LogP	Sulphur Frac	Solubility	H Donor Frac	Heteroatom Frac
1	Membrane Receptor	4,617	4,927	0.89	0.88	0.87	0.89	Aromatic Bonds Frac	sp2 Carbon Frac	Ringbonds Frac	Nitrogen Frac	Stereoatom Frac	Positive Atom Frac
2	7TM1	2,130	2,081	0.89	0.89	0.89	0.89	Rigidity Index	Carbon Frac	Polar SASA Frac	Num sp3 Carbons	Hydrogen Frac	Sp3 Carbon Frac
2	7TM2	119	107	1.00	0.88	0.90	1.00	sp2 Carbon Frac	Carbon Frac	Rigidity Index	Num Chains	SP3 Carbon Frac	Singlebonds Frac
2	7TM3	2,192	2,196	0.91	0.88	0.89	0.91	Num Chain Assemblies	Carbon Frac	LogP	Positive Atom Frac	Sp3 Carbon Frac	Cmp Zwitterion
2	Kinase	1,461	1,419	0.90	0.88	0.89	0.90	Oxygen Frac	Molecular SASA	Num Terminal Rotomers	Nitrogen Frac	Ringbonds Frac	Num Rings
2	LGIC	1,803	1,791	0.86	0.89	0.87	0.86	Sulphur Frac	Num Chains	Doublebonds Frac	Solubility	H acceptor Frac	Positive Atom Frac
2	TRP	43	39	1.00	1.00	1.00	1.00	Molecular Surface Area	Molecular PSA	Rotatable Bonds Frac	Num Terminal Rotomers	Ringbonds Frac	Rigidity Index
2	VGC	106	109	0.96	0.92	092	0.96	Ringbonds Frac	Num Halogens	Polar SASA Frac	Num Chains	Hydrogen Frac	Molecular Volume

Abbreviations: 7TM1 – Class A GPCRs, 7TM2 – Class B GPCRs, 7TM3 – Class C GPCRs, LGIC – Ligand Gated Ion Channels, TRP – Transient receptor potential channels, VGC – Voltage Gated Ion Channels, Frac – Fraction, Cmp – compound, H Acceptors – Hydrogen Bond Acceptors, H Donors – Hydrogen Bond Donors, LogD – distribution coefficient, LogP – partition coefficient, PSA – Polar Surface Area, SASA – Solvent Accessible Surface Area, sp2 – SP3 hybridized Carbons, sp3 – SP3 Hybridized Carbons, Num – Number of, n/a – Not Available.

Second to being able to predict if a compound will or will not be an allosteric modulator, it is also of interest to find out what properties are important to make this distinction. Given in Table 3 are the three most important properties that were correlated with the ‘allosteric’ class and the three most important properties that were correlated with the ‘non-allosteric’ class for each classification model. These properties allow the further investigation into what differentiates allosteric from non-allosteric compounds. While in most cases allosteric modulators were more lipophilic and non-allosteric compounds were associated with a higher polar surface area this was not always the case. Examples were the Transient Receptor Potential Channels (TRP) and Voltage Gated Ion Channels (VGC) target classes (L2 target class, ion channels), part of the Ion Channel (L1 target class). Here allosteric ligands had a larger polar surface area (TRP) or larger polar solvent accessible surface area (SASA) (VGC). Conversely non-allosteric ligands were more rigid (TRP). No explanation for this observation is currently available but possibly, in the case of these two ion channels, the uncompetitive binders could bind near the ion channel itself and hence resemble these ions that are transported by these proteins rather than resembling the natural regulators (which is Voltage in the case of VGC and can be diverse in the case of TRP). Note that this observation was absent for the Ligand Gated Ion Channels (LGIC) where the allosteric modulators seem to correspond more to what we observe in other protein classes (double bonds are favorable and solubility/positive atom fraction are not favorable). For the full table containing the results of all classification models trained on all targets in levels 0–2 (including class ‘undefined’ models) see supporting Table S6. In the final section the potential of the data set is demonstrated using three further different case studies.

### Case Study 2: HIV reverse transcriptase

To illustrate possible applications of the data set, the classification models were applied to a number of previously studied targets for which a range of allosteric inhibitors has been published. The first of these targets is the viral enzyme HIV-1 reverse transcriptase (HIV-RT), for which substantial SAR data and several approved drugs are well established [64], [65]. A relevant drug target in the treatment of HIV, this target will fall into ‘Enzyme’ L1 target class and is not further defined on lower target class levels due to the sparseness of other related proteins in version 14 of ChEMBL. Importantly, both allosteric and non-allosteric drugs have been successfully developed as therapeutics, and many co-crystal structures reported clarifying the binding sites of various compound classes, making this an ideal target case. Furthermore rational design and random screening have been used to extensively study the protein.

**Figure 7:**
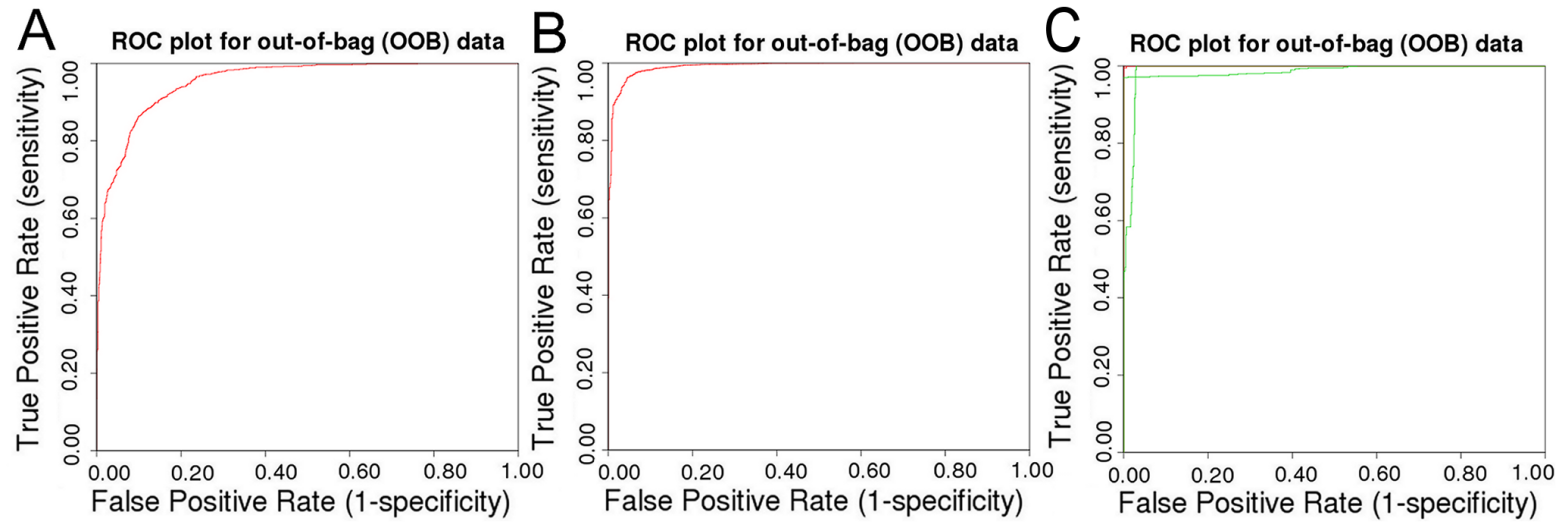
ROC curves for out-of-bag validation of the allosteric classifier models trained in case studies 2–4. (A) ROC curve for the HIV-RT classifier. (B) ROC curve for the adenosine receptors classifier. (C) ROC curve for the Protein Kinase B classifier (note that here a ternary model was used as opposed to a binary model).

Before training models the molecules were clustered based on FCFP_6 fingerprints. As can be expected there were some misclassifications in the dataset. Known allosteric compounds were in the non-allosteric training set (sharing scaffolds with known allosteric inhibitors). Moreover, a number of compounds in the allosteric training set were noted to be non-allosteric compounds (nucleotide like structures) and vice versa. Capturing this unannotated, or tacit knowledge within a field is challenging, and highlights some issues with data-mining the literature where ad hoc vocabularies and conventions are used; however, it also highlights the opportunity and added value for further curation. The clusters containing these compounds were reclassified based on the information in the original publications and subsequently a model was trained (Table 4). The model performed well with a sensitivity of 0.89, specificity of 0.88, PPV of 0.92, NPV of 0.84 and MCC of 0.76 and was hence interpreted (Figure 7).

**Table 4:** Overview of allosteric models used in the case studies.

Target	Type	MCC	Allosteric Recall (Sensitivity)	Non-Allosteric Recall (Specificity)	Allosteric Precision (PPV)	Non-Allosteric Precision (NPV)	Allosteric Biologicals Recall	Allosteric Biologicals Precision
HIV Reverse Transcriptase	Single target	0.76	0.89	0.88	0.92	0.84	n/a	n/a
Adenosine receptors	Target group	0.77	0.94	0.97	0.66	1.00	n/a	n/a
Protein Kinase B	Multiple allosteric classes	0.86	0.96	0.94	0.71	0.99	1.00	1.00

In the case of Protein Kinase B a three-class model was created (natural ligand mimicking peptides formed the third class). For class errors, sensitivity is recall of allosteric small molecules, specificity is recall of non-allosteric molecules, and in the case of protein kinase B a third class error (allosteric biological recall) is added. Likewise positive predictive value (PPV) quantifies precision for allosteric small molecules, negative predictive value (NPV) quantifies precision for non-allosteric molecules, and a third value quantifies precision for allosteric biological in the case of Protein Kinase B. In addition to these parameters, the MCC is calculated. Note that the values for the non-MCC parameters in the three-class model have been scaled to the same range as the binary classification models to allow direct comparison. Abbreviations: MCC – Matthews Correlation Coefficient, HIV – Human Immunodeficiency Virus.

The HIV-RT allosterism model showed the three most important descriptors for non-allosteric compounds to be fraction of Oxygen atoms as a part of all atoms (for instance the presence of a ribose moiety or a number of phospho groups contributes to this descriptor), a larger polar surface area and a larger fraction of atoms that are H-bond acceptors. Conversely, the following parameters were found to be predictive for allosteric ligands: a larger fraction of the bonds should be aromatic, the fraction of bonds that are ring-bonds should be higher and the distribution coefficient (LogD) should be higher (for a top 20 list see supporting Table S7). These results demonstrate that the here-published data set is a suitable starting point to create a model that can differentiate between likely non-allosteric and likely allosteric ligands for a specific target. However, after further data set curation this approach can lead to a well performing model that can reliably differentiate between these classes. This approach to developing a predictive method for allosterism is however not limited to enzymes as is shown in the following examples.

### Case Study 3: Adenosine receptors

Like HIV-RT, the class A GPCR adenosine receptors form a highly validated and important drug target, where both agonists and antagonists have a therapeutic potential. Moreover, there is now structural data for this GPCR target. Adenosine receptors are relevant targets in the treatment of diabetes and Parkinson's disease [66]. Allosteric modulation of the adenosine receptors has anticipated advantages over orthosteric modulation as it is expected to increase tissue specific selectivity and enable modulation of receptors present in the brain [66]. Moreover, class A GPCRs make up a large fraction of the targets present in ChEMBL. This is due to their high relative tractability, the historical research effort on this class, the large size (ca. 300 family members in the human genome), and linkage to many important diseases [4]. However, unlike HIV-RT no allosteric modulators of adenosine receptors have yet been launched as drugs. One compound, T-62, was under evaluation for the treatment of chronic pain but crashed out in phase 2 trials [67]. Moreover, there is a preclinical body of work that demonstrates allosteric modulation for these drug targets and hence they were chosen to be included here as a case study. Different from the HIV-RT case study is that here a group of closely related proteins is used rather than a single target. Hence it is shown that the current data set can also be used to capture properties that distinguish allosteric modulators for a family of targets.

Again some manual curation was needed before moving to model training. The main finding was the paper by Narlawar *et al.* [68]. This paper describes bitopic ligands that possess both allosteric and non-allosteric domains. The compounds were marked as allosteric due to the keywords noted in the abstract, yet the large non-allosteric part of the ligands (including a ribose moiety) deteriorates model performance. Similarly a number of ligands described by Jacobson *et al.* were included in the allosteric set as the abstract mentions that only some compounds appeared to bind at an allosteric site, yet the majority of the 78 compounds were non-allosteric, hence these were also cleaned [69].

The adenosine receptor allosteric modulator model performed well (sens 0.94; spec 0.97, PPV 0.66; NPV 1.00; and MCC 0.77; Figure 7), although the lower PPV lead us to believe further curation might improve model performance. The model was then interpreted. Allosteric ligands had a higher fraction of aromatic bonds, a higher LogD, and a higher average bond length compared to non-allosteric ligands. Whereas non-allosteric ligands had a higher heteroatom fraction and a larger polar surface area compared to allosteric ligands. Yet there was an interesting distinction with the HIV-RT models. The structures of known adenosine ligands (both allosteric and non-allosteric) are much more conserved than those of HIV-RT ligands. Hence structural features (in this case FCFP_6 substructures) were much more important in model creation compared to generic physicochemical properties (for example a xanthine scaffold was found to be correlated with non-allosteric modulators, supporting Table S8). Three substructures were shown to have high importance values in model creation (meaning that model quality significantly decreased by leaving them out of the descriptor set).

### Case Study 4: Kinase modulators, protein Kinase-B

A fourth and final case study presented in this paper is protein Kinase-B (PKB)/Akt 1. This enzyme target is relevant in oncology as it plays an important role in cellular survival pathways by inhibiting apoptotic processes [70], [71]. PKB differs from the previous targets as two different classes of allosteric modulators have appeared in the literature. As touched upon in the introduction, allosteric modulators of kinases can be small molecules that act for instance by shifting the balance of protein dynamics (e.g. locking a protein in an inactive conformation). However in the case of kinases where orthosteric modulators are defined as ATP-competitive, allosteric modulators can also be compounds that resemble the substrate of the kinase and hence be peptides (protein like compounds). In the current case study the allosteric modulators hence make up two major classes, one of which are large peptide like compounds. As such Protein Kinase B is an interesting target that forms the inverse of the class B GPCRs mentioned above. The non-allosteric modulators in this case were all ATP-competitive and it was hypothesized that this class forms a group that is more similar chemically than the allosteric modulators. Given the clear distinction between allosteric modulators that are peptidomimetic and small molecule allosteric modulators, the chosen course of action was to train the model using a three-class model rather than a binary classification model. The model had good predictivity (sens 0.96; spec 0.94; PPV 0.71; NPV 0.99; and MCC 0.86; Figure 7); the added third class, ‘allosteric biological’, was predicted very well with recall 1.00 and predictive value 1.00.

As expected, properties mostly related to size (Molecular polar surface area, volume) were correlated with the biological allosteric modulators as is the ChEMBL calculated molecular class ‘biological’. The physicochemical properties mostly correlated with small molecule allosteric modulators were number of chain assemblies, ringbond fraction, carbon fraction, and number of sp2 hybridized carbons. Additionally the ChEMBL calculated molecular class ‘small molecule’ was correlated to small molecule allosteric modulators. Interestingly, properties Lipinski pass, aromatic bonds frac, ringbonds frac, and LogD were also correlating with non-allosteric modulators (contrary to the trends observed in other targets). This is likely due to the fact that ‘small molecule allosteric modulators’ and ‘small molecule ATP competitive modulators’ more closely resemble each other than they do the ‘biological allosteric modulators’ in terms of physicochemical properties. Moreover the non-allosteric/ATP-competitive set contained a number of drugs, which are highly optimized structures. Yet, LogD, and ringbonds fraction correlated to both the allosteric and non-allosteric small molecule classes. Conversely, negative atom fraction and number of hydrogen bond acceptors were correlated with only non-allosteric compounds (likely due to the need for ATP-competitive compounds to also resemble parts of ATP), but this effect was less pronounced. Also in this case study (similar to HIV RT) sub-structural features were observed to be very important. Moreover, in the biological allosteric modulators class protein/peptide backbone fragments were appearing as important in combination with charged arginine side chains. Inversely, in the case of small molecule allosteric modulators the important substructures mostly contain aromatic rings. For a longer list see supporting Table S9.

### Prospective use of allosteric classifiers

In the case studies the potential of the data set identified and provided in this paper is demonstrated. The dataset is shown to be a solid starting point for allosteric focused drug discovery towards existing targets or towards new targets. With modest further curation highly predictive models could be obtained. While it is outside the scope of this paper to provide a case study on all potentially interesting protein targets, possible other examples included in the set are (but not limited to): Kinesin EG5 [72]–[74], Alcohol dehydrogenases (e.g. Isocitrate dehydrogenase 1 and 2 (ICDH)) [75], and class C GPCRs [26].

The models obtained here trained on the full allosteric modulator set should have a broad domain of applicability due to their generic nature (physicochemical properties were used as descriptors). Hence it is expected that these models are not limited to certain known chemical motifs as would be the case when using circular fingerprints. While also outside the scope of the current paper, the authors would very much welcome a prospective validation of the models. It should be noted that these models are solely classifying between ‘a likely allosteric interaction’ and ‘a likely non-allosteric interaction’. Hence the models cannot be used to predict the affinity of ligands on certain targets, but are able to predict the likely type of interaction for a given interaction. As such these models should ideally be *combined with* dedicated bioactivity models that can predict the affinity of molecules on a certain target and not *replace* them. Hence the allosteric classifiers can be used as a secondary filter when selecting compounds from a chemical vendor to be tested experimentally.

The authors feel that other potential applications could be the following: Firstly, creation of allosteric focused libraries based on known chemical properties of allosteric modulators, these libraries can be further sub divided on target type (e.g. Class A GPCR or Protein Kinase). Secondly, determination of interaction type of hits retrieved from HTS screening (allosteric or non-allosteric).

The authors are very open for potential collaborative projects to experimentally validate the approach as described here. Hence the authors would urge readers to contact them when they are interested in a specific set of allosteric modulators.

### Conclusions

As stated in the introduction, the term allosteric modulator is a very broad definition directly depending on the target (class) in question. Despite the presence of peptidic ligands and very diverse chemistry, there are some general conclusions that can be drawn from the current work.

Allosteric modulators tend to be more rigid and lipophilic structures compared to the background set. This is in line with their mode of action via binding in distinct structural locations of proteins rather than catalytic or agonist sites. Yet the magnitude of these changes in physicochemical properties depends on the target in question and the non-allosteric ligands. Moreover, it is observed that allosteric modulators are constrained to a narrower structure activity window than are non-allosteric modulators. When the physicochemical properties of allosteric modulators are compared to all ligands for a target, the allosteric modulators are often a subset of the non-allosteric ligands.

Secondly, it is observed that allosteric modulators are interesting drugs for several reasons. They tend to adhere better to Lipinski's rule of 5, making them good candidates for oral formulation. This could indicate that, if allosteric hits are identified for a target, allosteric ligands are more developable then non-allosteric ligands. Moreover, a trend is observed that allosteric modulators are less promiscuous than non-allosteric modulators.

Thirdly, the absolute potency for allosteric modulators is observed to be lower, while their binding efficiency and surface ligand efficiency is similar. Some potential causes are discussed here, but before a qualitative statement can be made about this observation further research is required. However this observation does call for the adaptation of screening assays to pick up the lower affinity compounds.

In conclusion, the differences between non-allosteric and allosteric modulators for a given target are usually such that it is not straightforward to turn a non-allosteric compound into an allosteric compound or vice versa. Yet it is these chemical differences that allow the creation of classification models that can distinguish between allosteric and non-allosteric modulators. These models are shown to perform better if the target definition is more concise, yet even without these constraints already predictive models were constructed. Hence non-allosteric and allosteric inhibition of a single target can be considered different target classes overall. The work performed here should lead to improvement of bioactivity models by providing tools to incorporate binding mode as a descriptor for compounds and hence reducing the noise present in a data set.

While the authors have demonstrated in the current paper how the dataset can be used as a starting point for allosteric drug design, full manual curation of the dataset is at the moment infeasible. Hence the authors encourage everybody who encounters an error or misclassification in this data set to contact them so that curation can take place via crowdsourcing and the quality of this data in ChEMBL can increase.

## Methods

### Data set

The data set was obtained from ChEMBL version 14 [31]. For the allosteric set, abstracts and titles of journal articles were searched for keywords (supporting Table S10). For hits both PubMed ID and citation information (primary author, year, journal, volume, and starting page) were kept. From these retrieved records the primary target (based on bioactivity annotation frequency for targets considered in the document) was included along with all compounds annotated on this primary target. As a final step duplicate compounds were removed for each target ID. Herein a distinction was made in the quality of the bioactivity measurement, best measurements (e.g. pKi) were favored over lower quality measurements (e.g. activity comment ‘active’). The background set was retrieved in a similar fashion, but here all document IDs that were not part of the allosteric set were kept. Finally, the balanced non-allosteric set was retrieved from the full non-allosteric set by keeping a random percentage of bioactivities from each L2 target class which was roughly equal in size to the number of bioactivities present in the allosteric set. All data is available on www.gjpvanwesten.nl/allosterism or ftp.ebi.ac.uk/pub/databases/chembl/Allosterism, see supporting Figure S4 for details.

### Compound pre-treatment

Compounds were standardized, charged at a pH of 7.4, salts were removed and 2D and 3D coordinates were calculated. All of this was done in Molsoft ICM version 3.7-2d [76].

### Compound descriptors

Volume, Polar Surface Area, Molecular weight, and drugLikeness were calculated in Molsoft ICM, carbon hybridization states were calculated using the Perl molecular toolkit in Pipeline Pilot [76], [77]. For partition coefficient (LogP) calculations it has been shown that consensus methods perform well [78], hence the used LogP value was the average of AlogP calculated in Pipeline Pilot, logP according to Molsoft ICM and ACD LogP [76], [77], [79]. Similarly LogD was the average of the Pipeline pilot module and ACD LogD, finally solubility was the average of the pipeline pilot calculator and Molsoft ICM value. The remaining compound physicochemical descriptors were calculated in Pipeline Pilot using the chemistry component collection [77]. The Lipinski Pass/Fail class was calculated allowing no violations. For the individual case studies additional FCFP_6 descriptors were used, on these Bayesian feature selection from Pipeline Pilot was applied to transfer them into a 512 bits fixed bitstring [77], [80].

Finally, the rigidity index was an estimation of compound rigidity that was calculated as follows: (AromaticBonds fraction)+(1-RotatableBonds fraction)+Aliphatic Ringbonds fraction+(1-SingleBonds fraction)+DoubleBonds fraction+TripleBonds fraction+BridgeBonds fraction)/7;

### Target pre-treatment

Target information from ChEMBL (Uniprot ID, target classification) was kept as it was defined in ChEMBL. However, when target classification levels were unpopulated the value was replaced with ‘Undefined’.

### Machine learning

Models were trained in Pipeline Pilot using the ‘Random Forest’ component. This component uses R-Statistics (version 2.15.0) and the ‘forest’ package [81], [82]. For variable importance selection permutation based selection and Gini importance without scaling were used, as recommended by Strobl *et al.* [83], [84]. Important variables were selected based on Pareto optimization of both importance values and class correlation values (e.g. correlation with ‘Allosteric’ class).

### Model validation

Validation was performed using 5 different metrics these were: sensitivity (allosteric recall, the fraction of true positives of the total number of allosteric compounds), specificity (non-allosteric recall, the fraction of true negatives of the total number of non-allosteric compounds), positive predictive value (allosteric precision, the fraction of true positives of the total number of compounds predicted to be allosteric modulators), negative predictive value (non-allosteric precision, the fraction of true negatives of the total number of compounds predicted to be non-allosteric modulators), and the Matthews correlation coefficient [85]. Given a confusion matrix were A represents an allosteric modulator classification and B represents non-allosteric modulator classification, Sensitivity is class A recall, and specificity is class B recall, whereas positive predictive value is class A precision and negative predictive value is class B precision (Table 5).

**Table 5:** Binary classification confusion matrix.

	Model Predicts A	Model Predicts B	
Experiment Measures A	AA	AB	Class A recall (Sensitivity) AA/(AA+AB)
Experiment Measures B	BA	BB	Class B recall (Specificity) BB/(BB+BA)
	Class A precision (PPV) AA/(AA+BA)	Class B precision (NPV) BB/(BB+AB)	

Recall values are calculated over the rows and precision values over the columns.

For the MCC equation (1) was used; herein the numerator is the product of the correctly predicted data points minus the product of the incorrectly predicted data points. The denominator is formed by the square root (2-classes) of the total product of all possible sums of correct and incorrectly predicted data points.
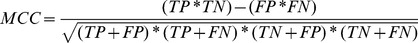
(1)


Note that false negatives are missed class A predictions and false positives are missed class B predictions. Hence this can be rewritten as follows:

Numerator:
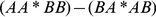
(2)


Denominator:
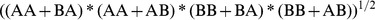
(3)


In the case of the three-class model (ternary classification) these calculations were modified to represent the three-class confusion matrix. Assume class A to be allosteric modulators, class B to be non-allosteric modulators and class C to be biological allosteric modulators. Sensitivity remains the fraction of true positives of the total number of allosteric compounds (here class A recall), specificity remains the fraction of true negatives of the total number of non-allosteric compounds (here class B recall), positive predictive value remains the fraction of true positive of the total number of compounds predicted to be allosteric modulators (here class A precision), and negative predictive value remains the fraction of true negatives of the total number of compounds predicted to be non-allosteric modulators (class B precision). Additionally a class C recall (the fraction of true allosteric-biological predictions of the total number of allosteric-biological compounds) and precision (the fraction of true allosteric-biologicals of the total number of compounds predicted to be allosteric biologicals) are introduced. It should also be noted that the baseline values for a random model in a ternary classification model are expected to be around 0.33 (33% correctly predicted compared to 66% incorrectly predicted). This is lower than the value of 0.50 (50% correct prediction and 50% incorrect prediction) for a binary model. Hence values were scaled to be directly comparable between the two model types.

Equation (1) was again used for the MCC but adapted to the ternary matrix (Table 6); the product of the correctly predicted data points minus the product of the incorrectly predicted data points forms the numerator. The denominator is formed by the cube root (3-classes) of the total product of all possible sums of correctly and incorrectly predicted data points.

**Table 6:** Ternary classification confusion matrix.

	Model Predicts A	Model Predicts B	Model Predicts C	
Experiment Measures A	AA	AB	AC	Class A recall AA/(AA+AB+AC)
Experiment Measures B	BA	BB	BC	Class B recall BB/(BA+BB+BC)
Experiment Measures C	CA	CB	CC	Class C recall CC/(CA+CB+CC)
	Class A precision AA/(AA+BA+CA)	Class B precision BB/(AB+BB+CB)	Class C precision CC/(AC+BC+CC)	

Recall values are calculated over the rows and precision values over the columns.

The following types are defined:

AB+AC = AX (Missed class A predictions)

BA+BC = BX (Missed class B predictions)

CA+CB = CX (Missed class C predictions)

Hence the MCC can be written as follows:

Numerator:
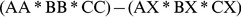
(4)


Denominator:
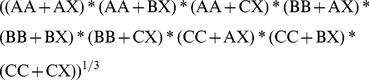
(5)

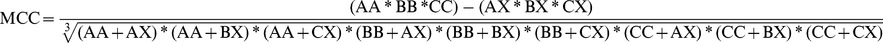
(6)


The MCC still produces values between 1 (perfect prediction), 0 (random prediction) and −1 (anti correlation) and need not be scaled, contrary to the recall values and predictive values as the full confusion matrix is considered in absolute numbers when calculating the MCC.

### Substructure frequency analysis

Substructures were obtained using pharmacophore feature class based circular fingerprints (FCFP_6) [63], [80]. For all present substructures, substructure frequencies were obtained from the full data set (background frequency), the allosteric set per L2 target (allosteric frequency), and the non-allosteric set per L2 target (non-allosteric frequency). These frequencies were normalized per set (substructure frequency as a fraction of the total substructures per set) to prevent a biased ranking. Subsequently all substructures were ranked based on their normalized frequency.

Enrichment was calculated based on the logarithm of the normalized ranks quotient (between allosteric and background or between non-allosteric and background). These final scores were ranked to obtain the final scored rank.

### Supporting Information available

4 supporting figures (Figure S1, S2, S3, S4) and 10 supporting tables (Table S1, S2, S3, S4, S5, S6, S7, S8, S9, S10) that further support the findings are available online. In addition, the datasets, further chemical analyses (per target level), physicochemical property histograms (for L0, L1, and L2), all model training and validation reports, and delimited text files are available online: www.gjpvanwesten.nl/allosterism or ftp.ebi.ac.uk/pub/databases/chembl/Allosterism.

## Supporting Information

Figure S1 The ChEMBL-14 target hierarchy; shown are the first three levels where L0 means the full allosteric versus the full non-allosteric set (protein binding compounds). Descending the hierarchy leads to a finer grained target classification, which eventually culminates in individual proteins (L8). The target distribution overview in the main text is made at target level L2 (red circle).
(TIF)

Figure S2 L1 target class distribution of both the allosteric (A) and non-allosteric data (B) sets. Also here the distribution of the target classes differed between the two sets.
(TIF)

Figure S3 Bar chart of all the mean values for all descriptors in both the allosteric and non-allosteric set of the 7TM2 class (Class B GPCRs). Note that delimited text files are available on www.gjpvanwesten.nl/allosterism or ftp://ftp.ebi.ac.uk/pub/databases/chembl/Allosterism.
(TIF)

Figure S4 Layout of the online ftp archive with the extra supporting information.
(TIF)

Table S1 Physicochemical descriptors used.
(DOC)

Table S2 Examples of positively enriched allosteric substructures class B GPCR ligands.
(TIF)

Table S3 Examples of negatively enriched allosteric substructures class B GPCR ligands.
(TIF)

Table S4 Examples of positively enriched non-allosteric substructures class B GPCR ligands.
(TIF)

Table S5 Model improvement when including fingerprints in model construction.
(DOCX)

Table S6 Allosteric models for balanced data sets of L0, L1, and L2 groups.
(DOCX)

Table S7 Top 20 property importance for the optimized HIV RT model.
(TIF)

Table S8 Top 20 property importance for the optimized adenosine model.
(TIF)

Table S9 Top 17 property importance for the optimized protein kinase B model.
(TIF)

Table S10 Keywords used to retrieve the allosteric set.
(DOCX)
