# Supplementary figures and images for: Chemical, Target, and Bioactive Properties of Allosteric Modulation
Source: PLoS Comput Biol. 2014 Apr 3;10(4):e1003559. doi: 10.1371/journal.pcbi.1003559 (PMC3974644; doi:10.1371/journal.pcbi.1003559)

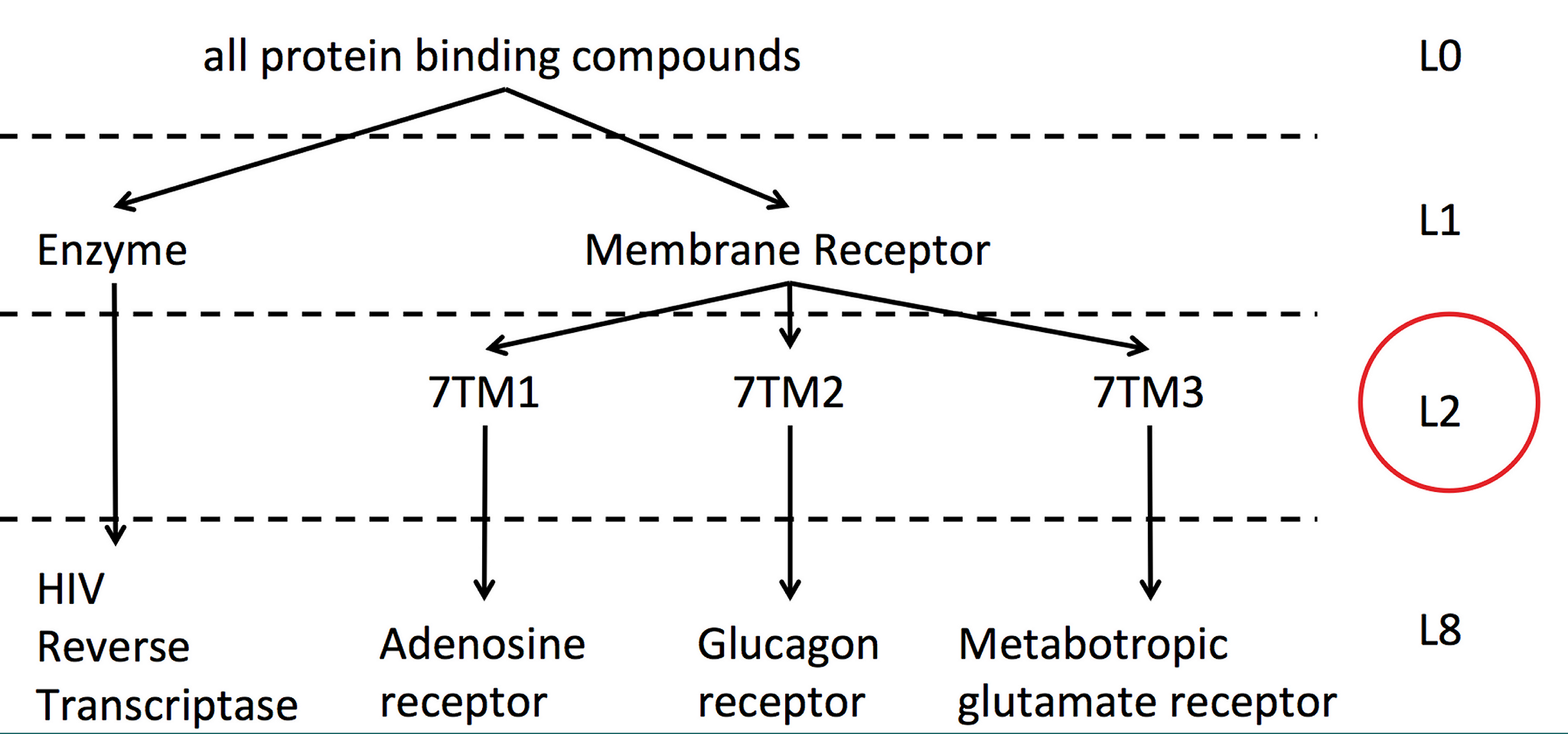

Supplement: Figure S1 — The ChEMBL-14 target hierarchy; shown are the first three levels where L0 means the full allosteric versus the full non-allosteric set (protein binding compounds). Descending the hierarchy leads to a finer grained target classification, which eventually culminates in individual proteins (L8). The target distribution overview in the main text is made at target level L2 (red circle). (TIF) [file pcbi.1003559.s001.tif]

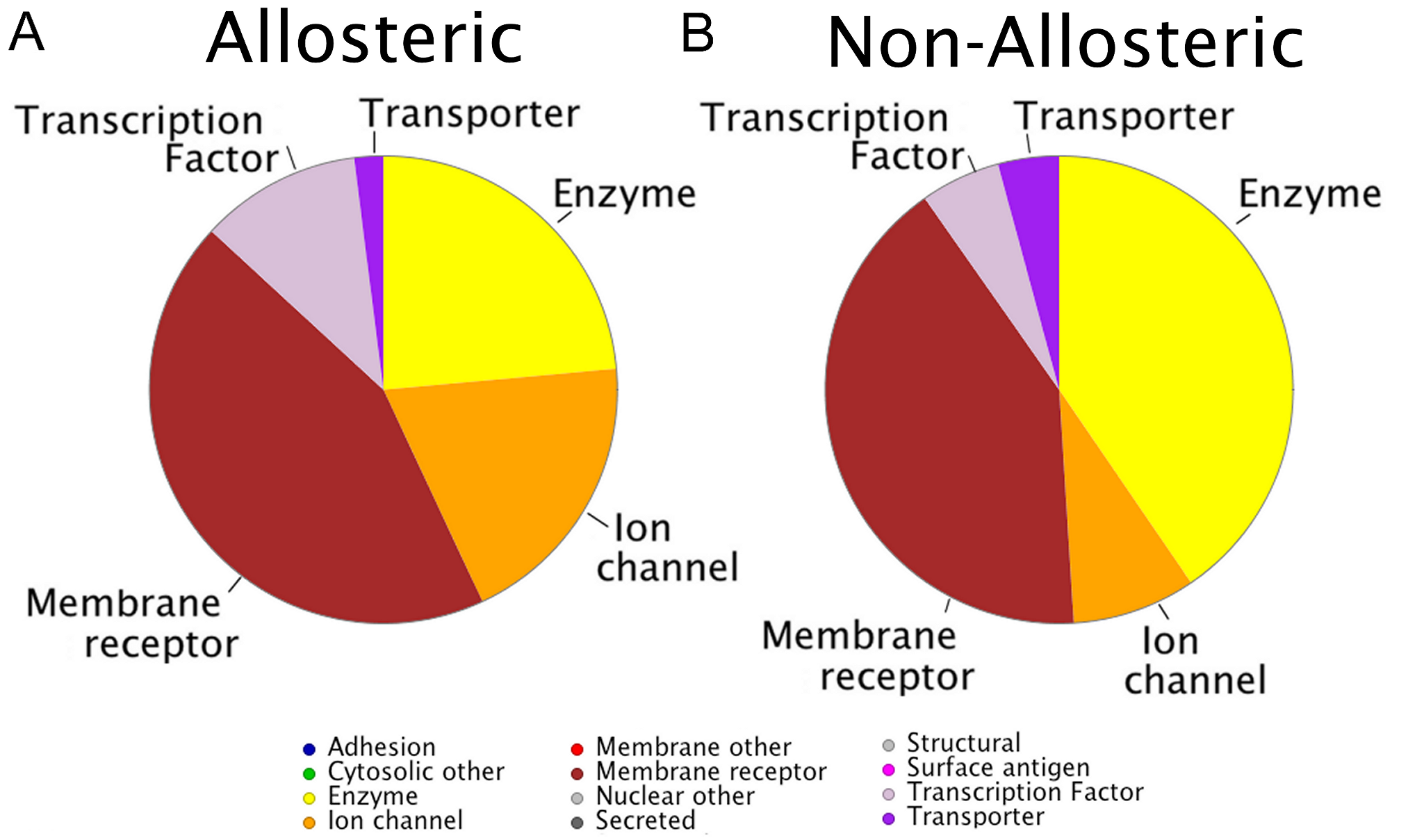

Supplement: Figure S2 — L1 target class distribution of both the allosteric (A) and non-allosteric data (B) sets. Also here the distribution of the target classes differed between the two sets. (TIF) [file pcbi.1003559.s002.tif]

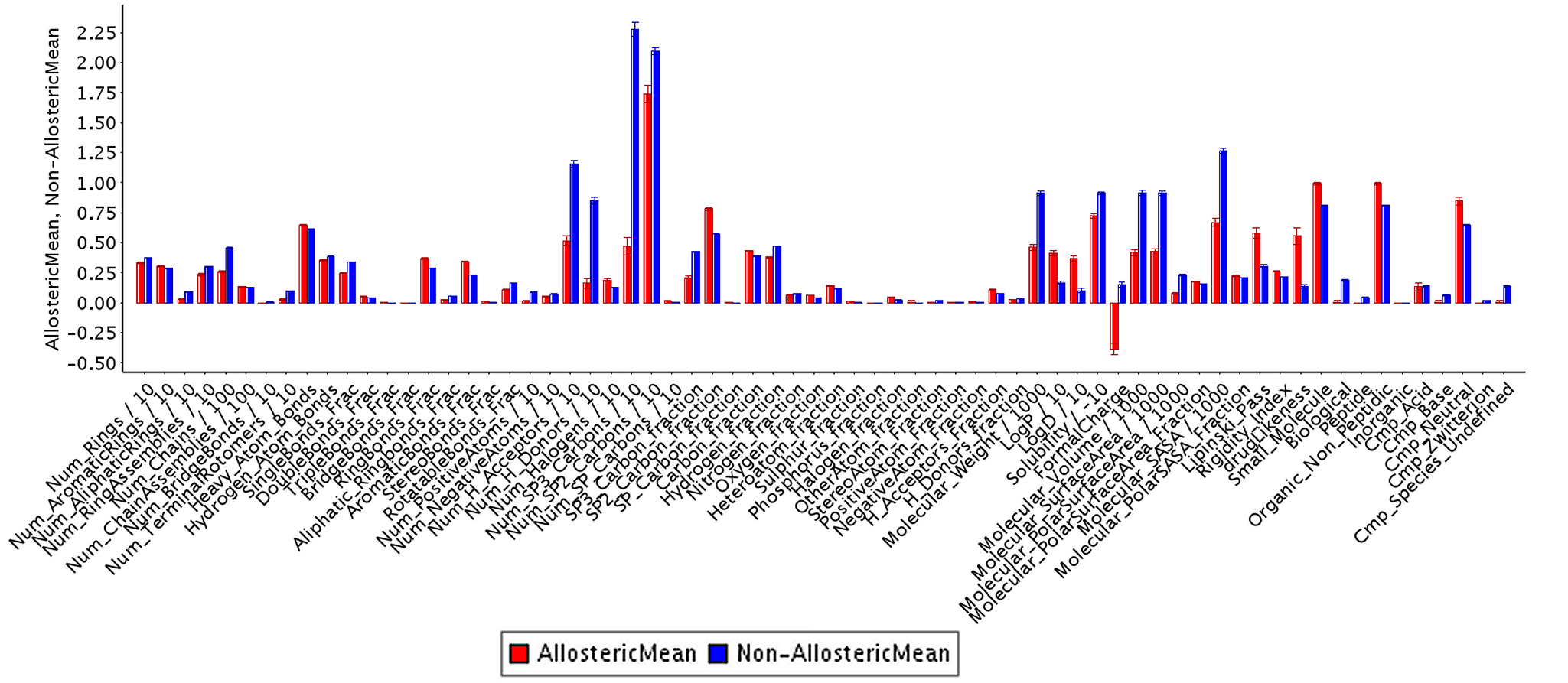

Supplement: Figure S3 — Bar chart of all the mean values for all descriptors in both the allosteric and non-allosteric set of the 7TM2 class (Class B GPCRs). Note that delimited text files are available on www.gjpvanwesten.nl/allosterism or ftp://ftp.ebi.ac.uk/pub/databases/chembl/Allosterism. (TIF) [file pcbi.1003559.s003.tif]

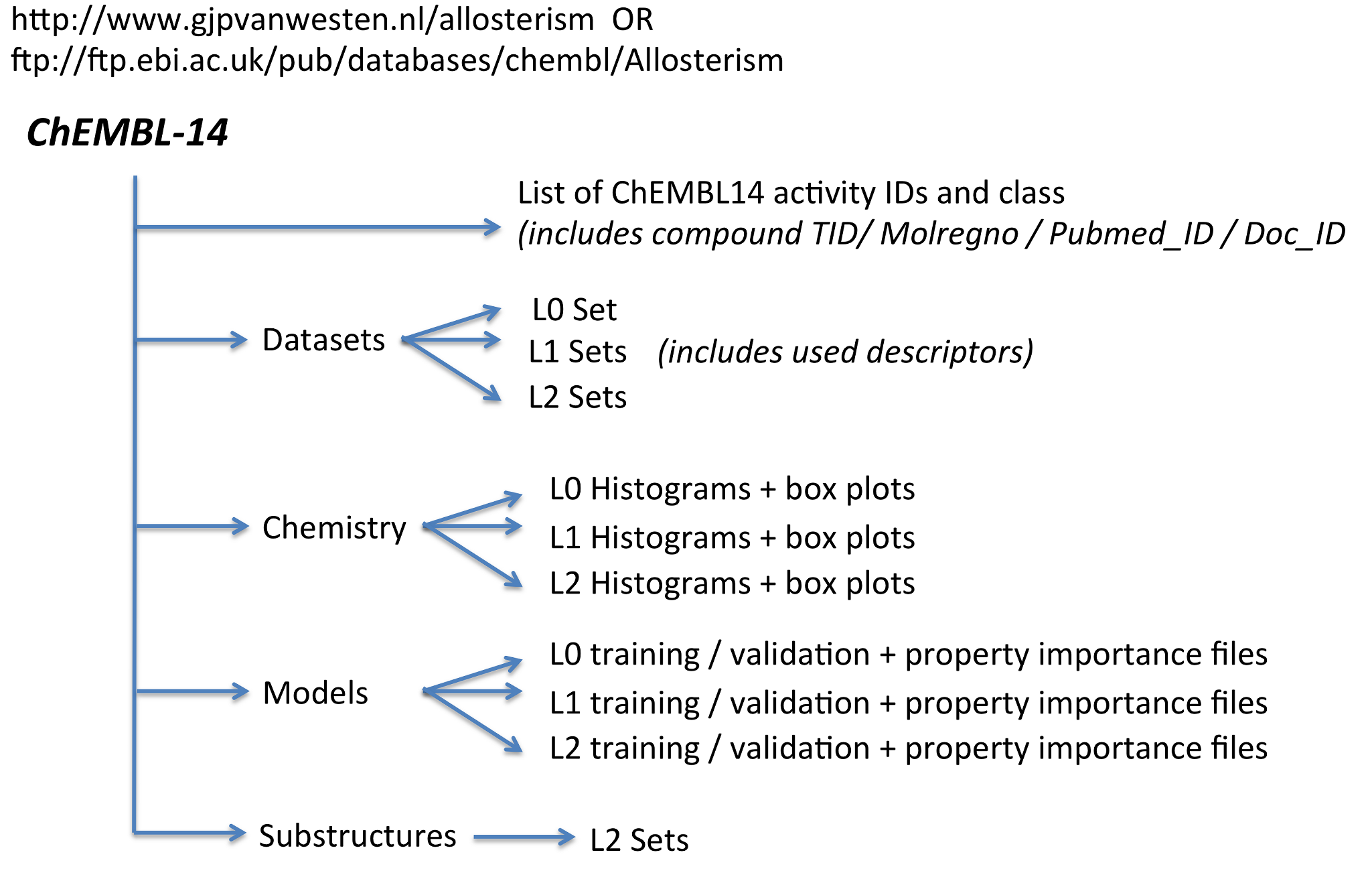

Supplement: Figure S4 — Layout of the online ftp archive with the extra supporting information. (TIF) [file pcbi.1003559.s004.tif]

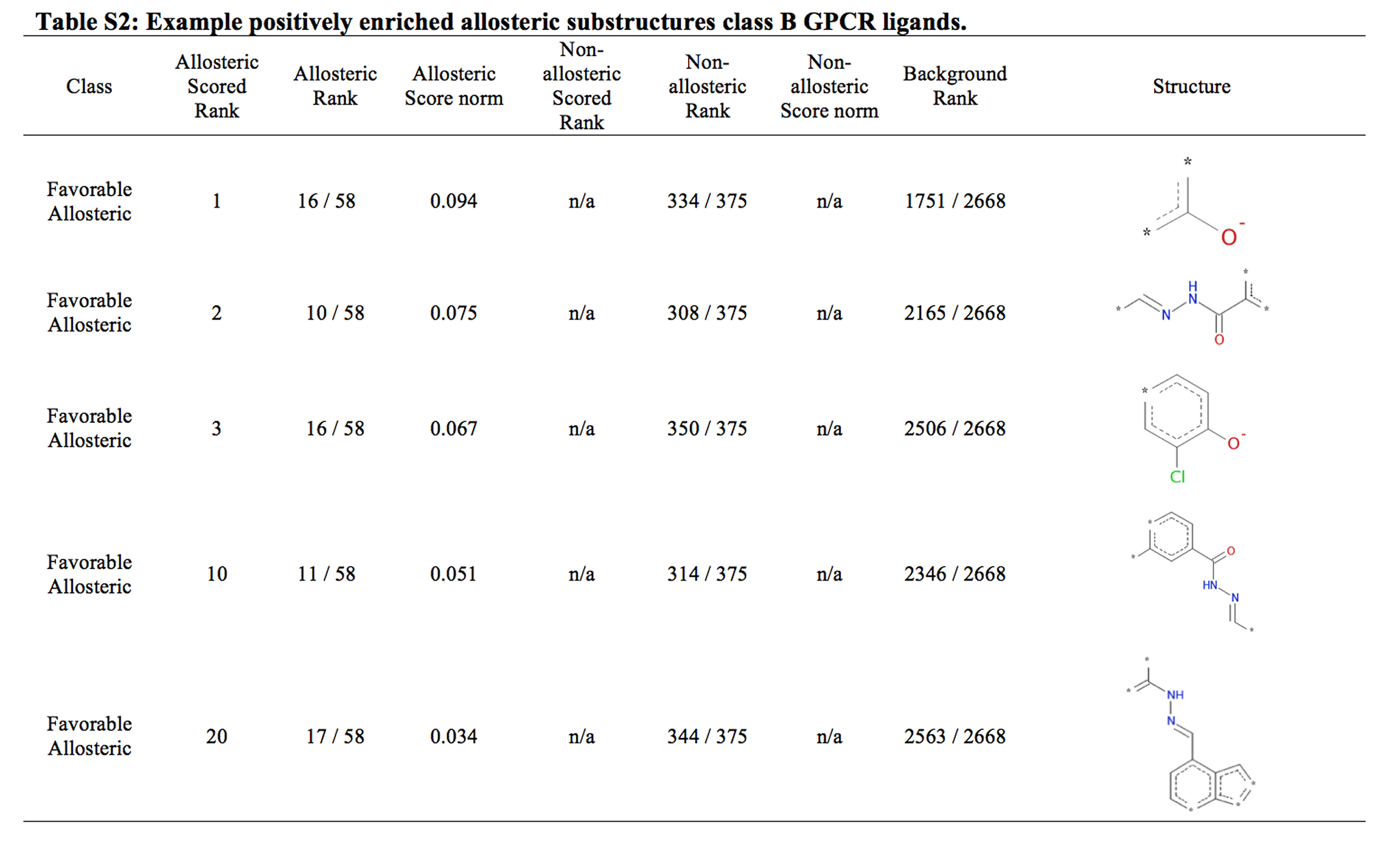

Supplement: Table S2 — Examples of positively enriched allosteric substructures class B GPCR ligands. (TIF) [file pcbi.1003559.s006.tif]

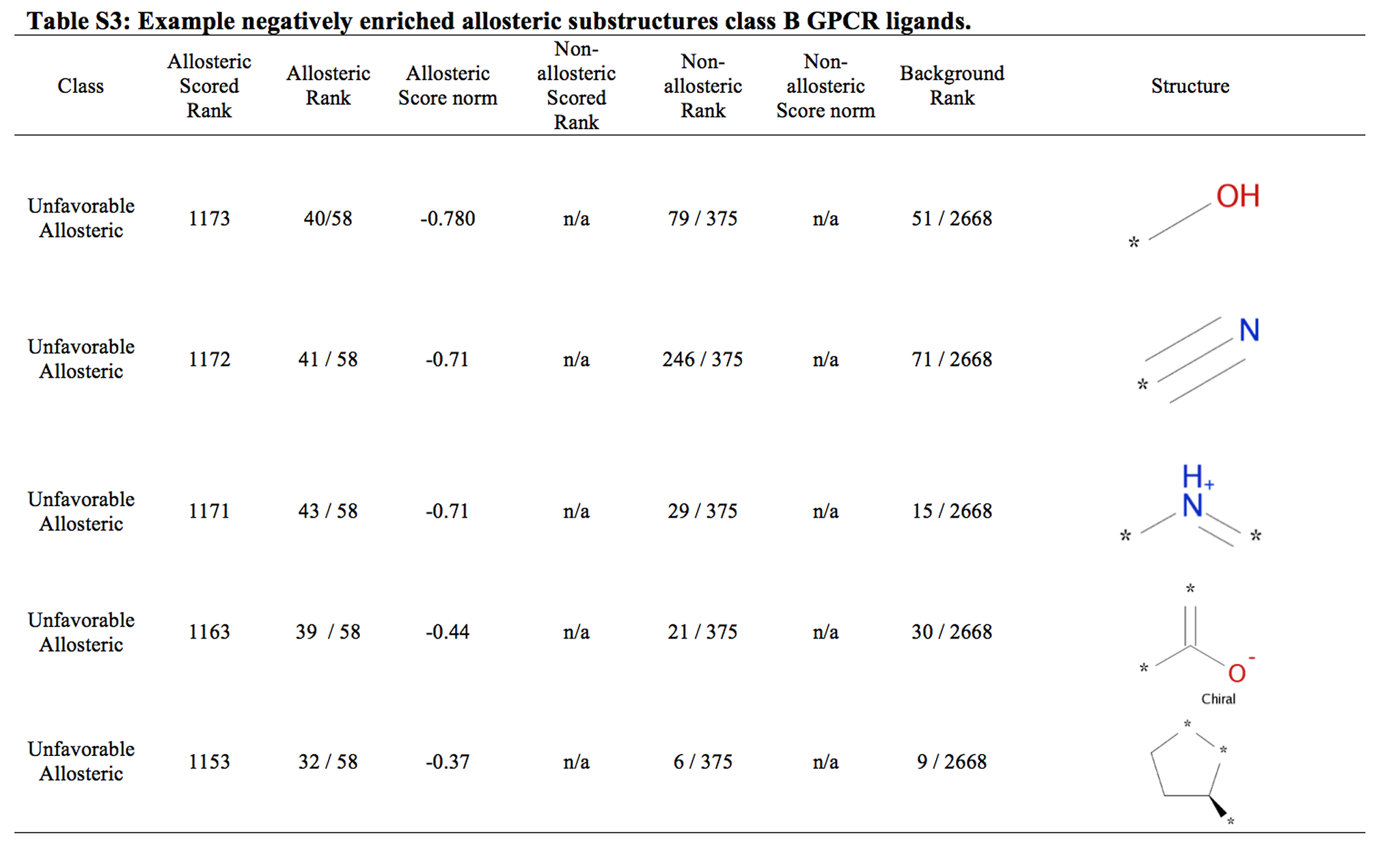

Supplement: Table S3 — Examples of negatively enriched allosteric substructures class B GPCR ligands. (TIF) [file pcbi.1003559.s007.tif]

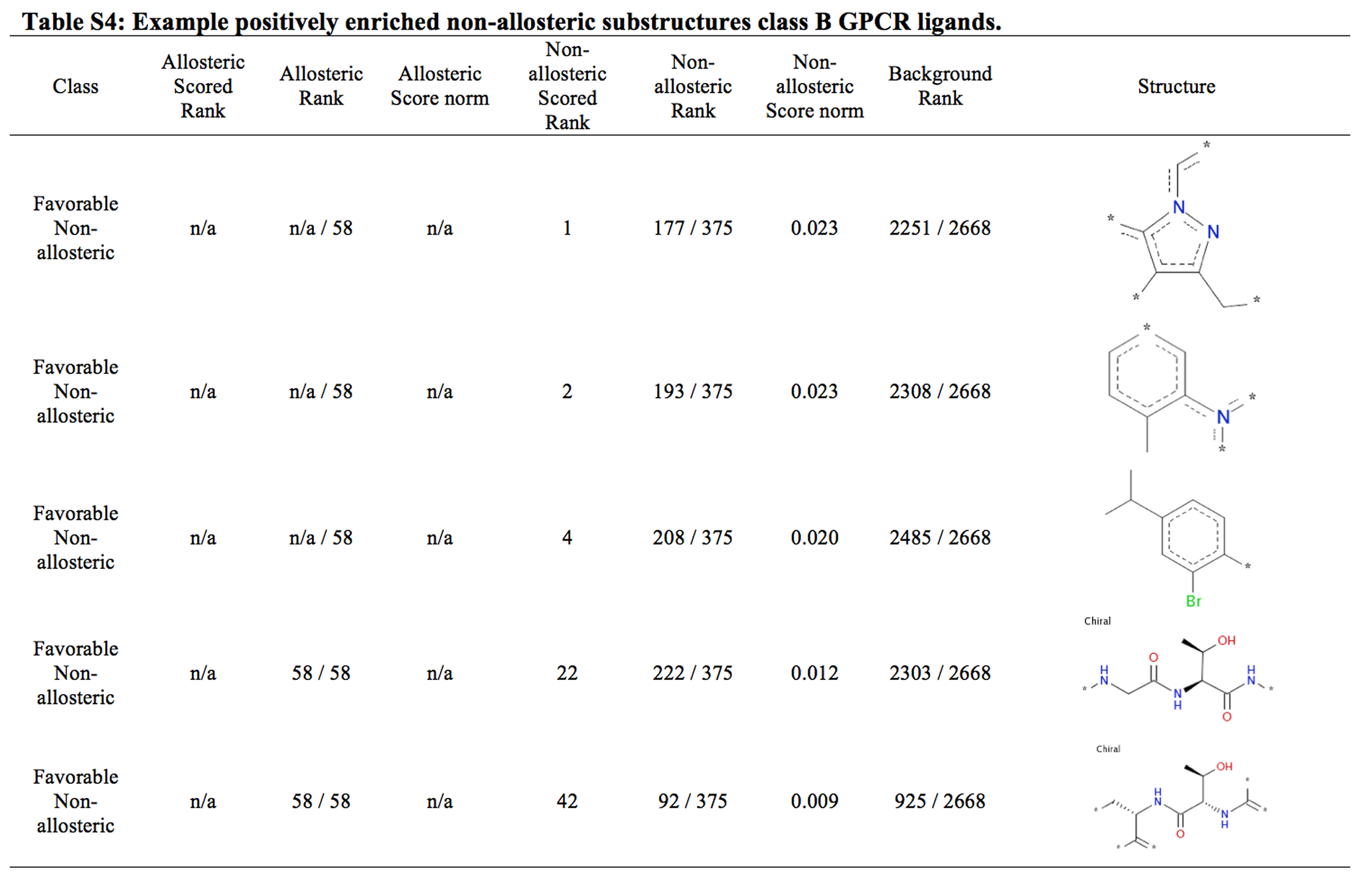

Supplement: Table S4 — Examples of positively enriched non-allosteric substructures class B GPCR ligands. (TIF) [file pcbi.1003559.s008.tif]

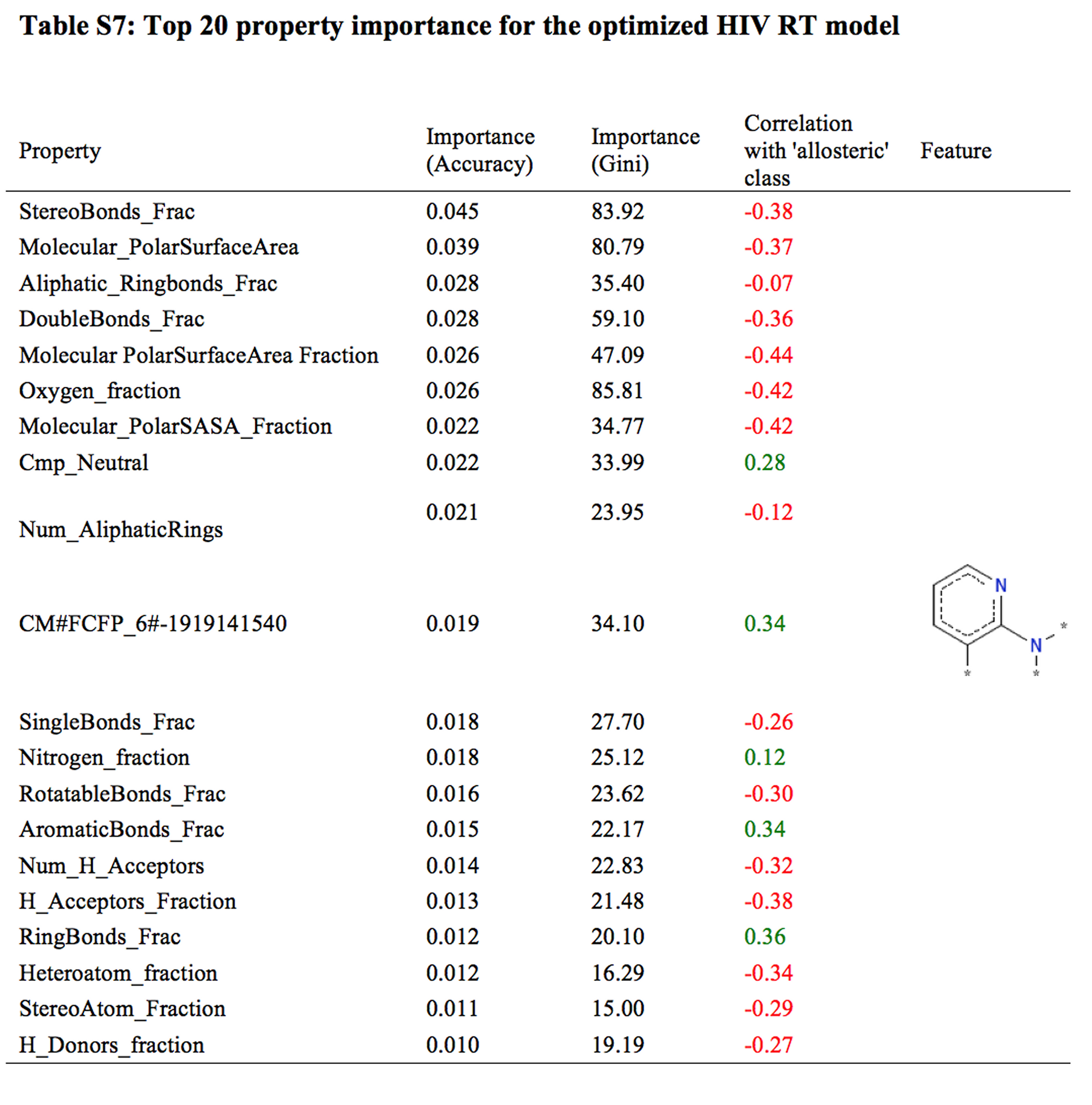

Supplement: Table S7 — Top 20 property importance for the optimized HIV RT model. (TIF) [file pcbi.1003559.s011.tif]

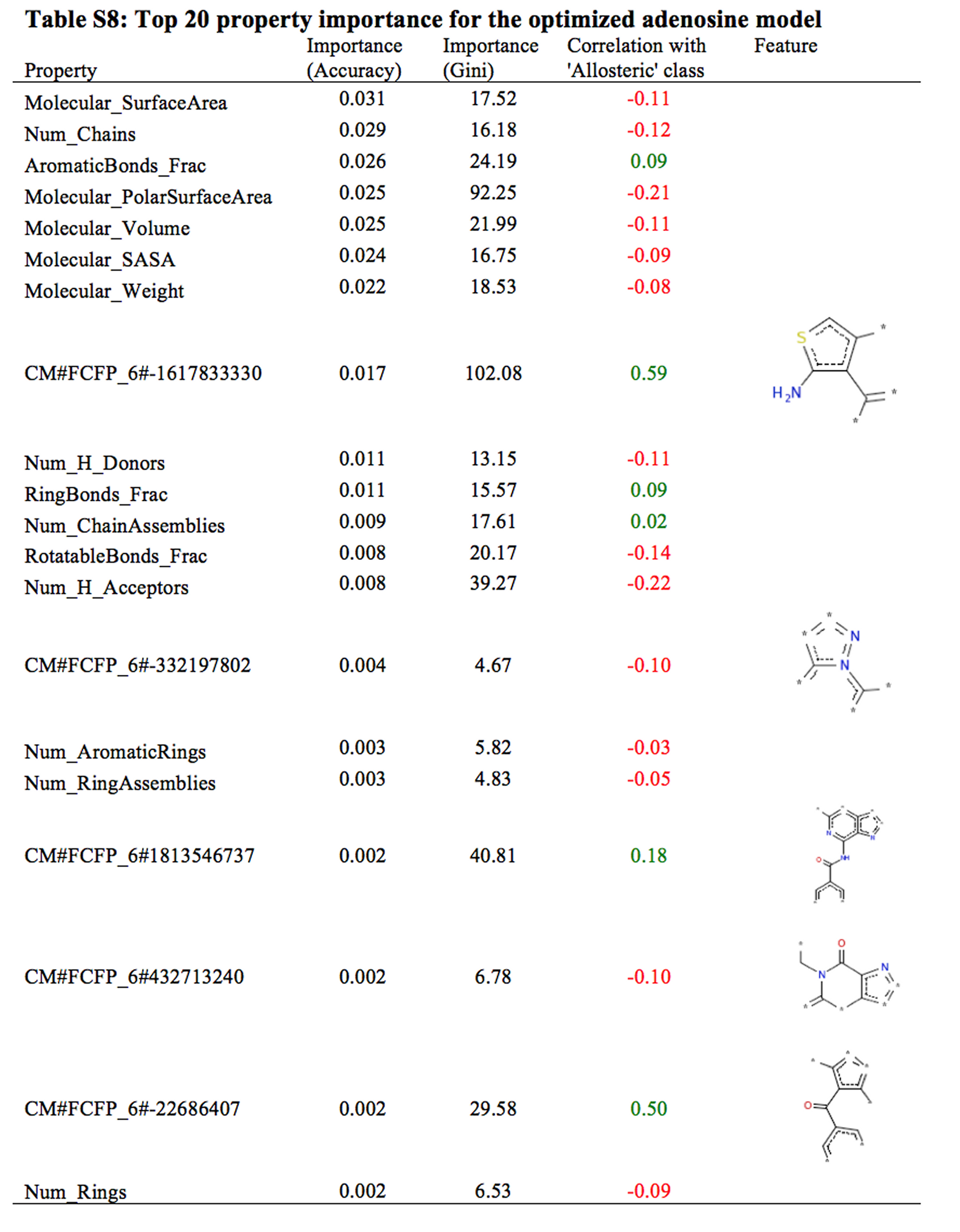

Supplement: Table S8 — Top 20 property importance for the optimized adenosine model. (TIF) [file pcbi.1003559.s012.tif]

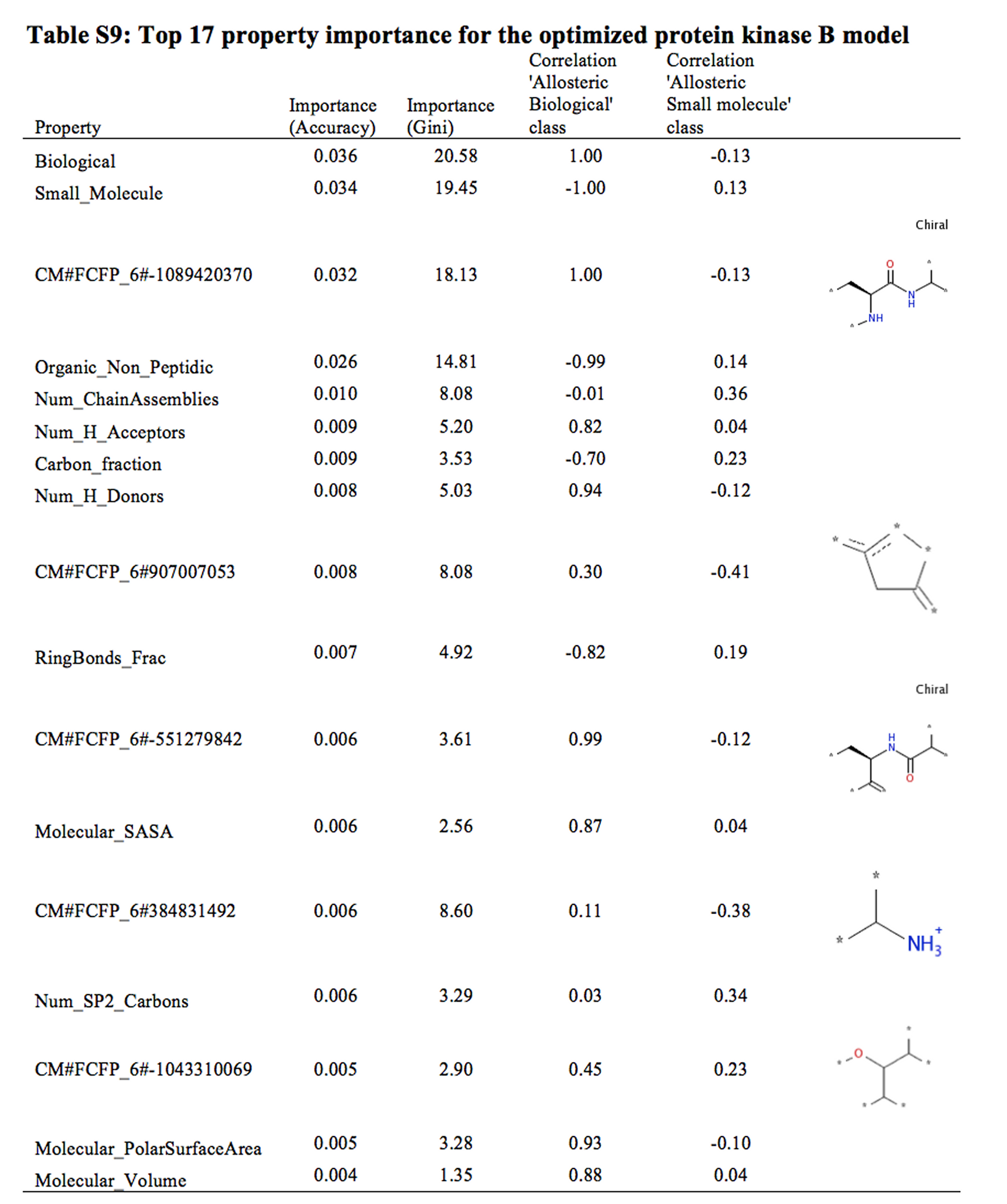

Supplement: Table S9 — Top 17 property importance for the optimized protein kinase B model. (TIF) [file pcbi.1003559.s013.tif]
