# Supplementary material for: Chemical, Target, and Bioactive Properties of Allosteric Modulation
Source: PLoS Comput Biol. 2014 Apr 3;10(4):e1003559. doi: 10.1371/journal.pcbi.1003559 (PMC3974644; doi:10.1371/journal.pcbi.1003559)
Supplement: Table S1 — Physicochemical descriptors used. (DOC) [file pcbi.1003559.s005.doc]

# Table S1: Physicochemical descriptors used

| Descriptor | Index | Description |
| --- | --- | --- |
| Num_Rings | 1 | Number of rings in the molecule |
| Num_AromaticRings | 2 | Number of aromatic rings |
| Num_AliphaticRings | 3 | Number of non-aromatic rings (2 - 1) |
| Num_RingAssemblies | 4 | Number of fragments after removing non-ring bonds |
| Num_Chains | 5 | ﻿Unbranched chains needed to cover all the non-ring bonds in the molecule |
| Num_ChainAssemblies | 6 | Number of fragments after removing Rings |
| Num_BridgeBonds | 7 | Bonds in bridgehead systems (﻿any rings that share more than one bond in common) |
| Num_TerminalRotomers | 8 | A non-terminal sp3 atom connected to three terminal atoms of the same type, or a non-terminal sp2 atom connected to two terminal atoms of the same type |
| Heavy_Atom_Bonds | 9 | Bonds between heavy atoms |
| Hydrogen_Atom_Bonds | 10 | Bond to hydrogen atoms |
| SingleBonds_Frac | 11 | Fraction of total bonds that are single |
| DoubleBonds_Frac | 12 | Fraction of total bonds that are double |
| TripleBonds_Frac | 13 | Fraction of total bonds that are triple |
| BridgeBonds_Frac | 14 | Fraction of total bonds that is a bridgebond |
| RingBonds_Frac | 15 | Fraction of total bonds in ring systems |
| Aliphatic_Ringbonds_Frac | 16 | Fraction of total bonds in a non aromatic ring |
| AromaticBonds_Frac | 17 | Fraction of total bonds in an aromatic ring |
| StereoBonds_Frac | 18 | Fraction of total bonds that are marked CisBondStereo, TransBondStereo, or UnknownBondStereo |
| RotatableBonds_Frac | 19 | Fraction of total bonds that ﻿are single bonds between heavy atoms that are both not in a ring and not terminal (special case amide C-N bonds are not rotatable) |
| Num_PositiveAtoms | 20 | Number of Atoms with a positive charge. |
| Num_NegativeAtoms | 21 | Number of Atoms with a negative charge. |
| Num_H_Acceptors | 22 | ﻿Heteroatoms (O, N, S, or P) with one or more lone pairs, excluding atoms with positive formal charges, amide and pyrrole-type N, and aromatic O and S atoms in heterocyclic rings |
| Num_H_Donors | 23 | ﻿Heteroatoms (O, N, S, or P) with one or more attached H atoms |
| Num_Halogens | 24 | Sum of F, CL, I and Br |
| Num_SP3_Carbons | 25 | Number of SP3 hybridized carbon atoms |
| Num_SP2_Carbons | 26 | Number of SP2 hybridized carbon atoms |
| Num_SP_Carbons | 27 | Number of SP hybridized carbon atoms |
| SP3_Carbon_Fraction | 28 | Fraction of total carbons that is SP3 hybridized |
| SP2_Carbons_Fraction | 29 | Fraction of total carbons that is SP2 hybridized |
| SP_Carbon_Fraction | 30 | Fraction of total carbons that is SP hybridized |
| Carbon_fraction | 31 | Fraction of atoms that is C |
| Hydrogen_fraction | 32 | Fraction of atoms that is H |
| Nitrogen_fraction | 33 | Fraction of atoms that is N |
| Oxygen_fraction | 34 | Fraction of atoms that is O |
| Heteroatom_fraction | 35 | Fraction of atoms that is O,N,S, or P |
| Sulphur_fraction | 36 | Fraction of atoms that is S |
| Phosphorus_fraction | 37 | Fraction of atoms that is P |
| Halogen_fraction | 38 | Fraction of atoms that is F, Cl, I, or Br |
| OtherAtom_fraction | 39 | Fraction of atoms that is not one of the above |
| StereoAtom_Fraction | 40 | Fraction of atoms marked ﻿EvenAtomStereo, OddAtomStereo, or UnknownAtomStereo |
| PositiveAtom_Fraction | 41 | Fraction of atoms with a positive charge |
| NegativeAtom_Fraction | 42 | Fraction of atoms with a negative charge |
| H_Acceptors_Fraction | 43 | Fraction of atoms that is hydrogen bond acceptor |
| H_Donors_fraction | 44 | Fraction of atoms that is hdyrogen bond donor |
| Molecular_Weight | 45 | Molecular weight (calc Molsoft ICM [76]) |
| LogP | 46 | Average value of ACD LogP, Accelrys AlogP and Molsoft ICM logP [76,77,79] |
| LogD | 47 | Average value of ACD LogD, Accelrys LogD at pH 7.4 [77,79] |
| Solubility | 48 | Average value of Accelrys solubility and molsoft solubility [76,77] |
| FormalCharge | 49 | Formal charge of the molecule at pH 7.4 (ionized in Molsoft ICM [76]) |
| Molecular_Volume | 50 | Molecular volume (calculated in Molsoft ICM [76]) |
| Molecular_SurfaceArea | 51 | Molecular Surface Area (2D Method) |
| Molecular_PolarSurfaceArea | 52 | Polar surface area (Molsoft ICM [76]) |
| Molecular_PolarSurfaceArea_Fraction | 53 | Fraction of total surface area that is polar |
| Molecular_SASA | 54 | Solvent Accessible Surface Area |
| Molecular_PolarSASA_Fraction | 55 | Fraction of SASA that is polar |
| Lipinski_Pass | 56 | N_Count + o_Count <= 10, MW <= 500, H_donors <= 5, AlogP <=5 |
| Rigidity_Index | 57 | ﻿(AromaticBonds_Frac + (1-RotatableBonds_Frac) + Aliphatic_Ringbonds_Frac + (1-SingleBonds_Frac) + DoubleBonds_Frac + TripleBonds_Frac + BridgeBonds_Frac) / 7 |
| drugLikeness | 58 | Druglikenes (calculated in Molsoft ICM [76]) |
| Small_Molecule | 59 | Binary flag for molecule class as calculated in ChEMBL |
| Biological | 60 | Binary flag for molecule class as calculated in ChEMBL |
| Peptide | 61 | Binary flag for molecule class as calculated in ChEMBL |
| Organic_Non_Peptidic | 62 | Binary flag for molecule class as calculated in ChEMBL |
| Inorganic | 63 | Binary flag for molecule class as calculated in ChEMBL |
| Cmp_Acid | 64 | Binary flag for ACD Molecular species |
| Cmp_Base | 65 | Binary flag for ACD Molecular species |
| Cmp_Neutral | 66 | Binary flag for ACD Molecular species |
| Cmp_Zwitterion | 67 | Binary flag for ACD Molecular species |
| Cmp_Species_Undefined | 68 | ACD Molecular species unknown / cannot be calculated |
