# Supplementary material for: Chemical, Target, and Bioactive Properties of Allosteric Modulation
Source: PLoS Comput Biol. 2014 Apr 3;10(4):e1003559. doi: 10.1371/journal.pcbi.1003559 (PMC3974644; doi:10.1371/journal.pcbi.1003559)
Supplement: Table S5 — Model improvement when including fingerprints in model construction. (DOCX) [file pcbi.1003559.s009.docx]

# Table S5: Model improvement when including fingerprints in model construction.

| Improvement | Class | | With Fingerprints | | | | | |  | | Without Fingerprints | | | | | |
| --- | --- | --- | --- | --- | --- | --- | --- | --- | --- | --- | --- | --- | --- | --- | --- | --- |
| (%) |  | Sens. | | Spec. | PPV | NPV | F | MCC |  | Sens. | | Spec. | PPV | NPV | F | MCC |
| **0.05** | **Overall Improvement** |  | |  |  |  |  |  |  |  | |  |  |  |  |  |
| *0.07* | *L0-Global* | *0.89* | | *0.88* | *0.89* | *0.89* | *0.89* | *0.77* |  | *0.83* | | *0.82* | *0.83* | *0.83* | *0.83* | *0.65* |
| 0.14 | L1-adhesion | 1.00 | | 1.00 | 1.00 | 1.00 | 1.00 | 1.00 |  | 1.00 | | 0.91 | 0.67 | 1.00 | 0.80 | 0.78 |
| 0.06 | L1-cytosolic other | 0.96 | | 0.92 | 0.91 | 0.96 | 0.93 | 0.87 |  | 0.88 | | 0.89 | 0.88 | 0.89 | 0.88 | 0.77 |
| 0.00 | L1-enzyme | 0.84 | | 0.82 | 0.84 | 0.82 | 0.84 | 0.66 |  | 0.84 | | 0.81 | 0.83 | 0.82 | 0.84 | 0.65 |
| 0.02 | L1-ion channel | 0.87 | | 0.91 | 0.90 | 0.87 | 0.88 | 0.77 |  | 0.86 | | 0.88 | 0.88 | 0.86 | 0.87 | 0.74 |
| 0.02 | L1-membrane recept. | 0.90 | | 0.90 | 0.89 | 0.90 | 0.89 | 0.79 |  | 0.89 | | 0.88 | 0.87 | 0.89 | 0.88 | 0.76 |
| 0.05 | L1-secreted | 0.90 | | 0.97 | 0.96 | 0.93 | 0.93 | 0.88 |  | 0.83 | | 0.95 | 0.92 | 0.88 | 0.87 | 0.79 |
| 0.03 | L1-transcription factor | 0.92 | | 0.88 | 0.88 | 0.92 | 0.90 | 0.80 |  | 0.89 | | 0.85 | 0.86 | 0.89 | 0.88 | 0.75 |
| 0.03 | L1-transporter | 0.87 | | 0.83 | 0.85 | 0.85 | 0.86 | 0.69 |  | 0.82 | | 0.83 | 0.84 | 0.81 | 0.83 | 0.65 |
| 0.04 | L1-undefined | 0.86 | | 0.96 | 0.93 | 0.92 | 0.90 | 0.84 |  | 0.83 | | 0.94 | 0.89 | 0.90 | 0.86 | 0.78 |
| *0.04* | *L1-Global* | *0.90* | | *0.91* | *0.91* | *0.91* | *0.90* | *0.81* |  | *0.87* | | *0.88* | *0.85* | *0.88* | *0.86* | *0.74* |
| 0.04 | L2-7tm1 | 0.92 | | 0.93 | 0.93 | 0.92 | 0.92 | 0.85 |  | 0.89 | | 0.89 | 0.89 | 0.89 | 0.89 | 0.78 |
| 0.02 | L2-7tm2 | 1.00 | | 0.92 | 0.93 | 1.00 | 0.96 | 0.92 |  | 1.00 | | 0.88 | 0.90 | 1.00 | 0.95 | 0.89 |
| 0.03 | L2-7tm3 | 0.93 | | 0.92 | 0.92 | 0.93 | 0.92 | 0.85 |  | 0.91 | | 0.88 | 0.89 | 0.91 | 0.90 | 0.80 |
| 0.05 | L2-cytochrome p450 | 0.95 | | 0.78 | 0.81 | 0.94 | 0.87 | 0.74 |  | 0.90 | | 0.75 | 0.78 | 0.88 | 0.84 | 0.66 |
| 0.00 | L2-electrochemical | 0.91 | | 0.90 | 0.91 | 0.90 | 0.91 | 0.81 |  | 0.96 | | 0.85 | 0.88 | 0.94 | 0.92 | 0.82 |
| 0.02 | L2-kinase | 0.91 | | 0.91 | 0.91 | 0.91 | 0.91 | 0.82 |  | 0.90 | | 0.88 | 0.89 | 0.90 | 0.90 | 0.79 |
| 0.02 | L2-lgic | 0.87 | | 0.91 | 0.90 | 0.88 | 0.89 | 0.78 |  | 0.86 | | 0.89 | 0.89 | 0.86 | 0.87 | 0.75 |
| 0.05 | L2-ntpase | 1.00 | | 0.88 | 0.89 | 1.00 | 0.94 | 0.88 |  | 0.96 | | 0.83 | 0.85 | 0.95 | 0.90 | 0.80 |
| 0.02 | L2-nuclear receptor | 0.91 | | 0.86 | 0.87 | 0.90 | 0.89 | 0.77 |  | 0.90 | | 0.84 | 0.86 | 0.88 | 0.88 | 0.74 |
| 0.06 | L2-phosphatase | 0.86 | | 0.95 | 0.95 | 0.86 | 0.91 | 0.81 |  | 0.86 | | 0.84 | 0.86 | 0.84 | 0.86 | 0.71 |
| 0.00 | L2-phosphodiesterase | 0.75 | | 0.71 | 0.75 | 0.71 | 0.75 | 0.46 |  | 0.75 | | 0.71 | 0.75 | 0.71 | 0.75 | 0.46 |
| 0.03 | L2-protease | 0.93 | | 0.87 | 0.89 | 0.93 | 0.91 | 0.81 |  | 0.88 | | 0.87 | 0.88 | 0.87 | 0.88 | 0.75 |
| 0.25 | L2-ryr | 0.50 | | 1.00 | 1.00 | 0.75 | 0.67 | 0.61 |  | 0.50 | | 0.67 | 0.50 | 0.67 | 0.50 | 0.17 |
| 0.00 | L2-sur | 1.00 | | 1.00 | 1.00 | 1.00 | 1.00 | 1.00 |  | 1.00 | | 1.00 | 1.00 | 1.00 | 1.00 | 1.00 |
| 0.00 | L2-trp | 1.00 | | 1.00 | 1.00 | 1.00 | 1.00 | 1.00 |  | 1.00 | | 1.00 | 1.00 | 1.00 | 1.00 | 1.00 |
| 0.01 | L2-undefined | 0.84 | | 0.87 | 0.87 | 0.84 | 0.85 | 0.71 |  | 0.84 | | 0.85 | 0.85 | 0.84 | 0.84 | 0.69 |
| -0.05 | L2-vgc | 0.96 | | 0.83 | 0.85 | 0.95 | 0.90 | 0.80 |  | 0.96 | | 0.92 | 0.92 | 0.96 | 0.94 | 0.88 |
| *0.03* | *L2-Global* | *0.90* | | *0.89* | *0.90* | *0.91* | *0.89* | *0.80* |  | *0.89* | | *0.86* | *0.86* | *0.89* | *0.87* | *0.74* |
| Improvement | Class | Sens. | | Spec. | PPV | NPV | F | MCC |  | Sens. | | Spec | PPV | NPV | F | MCC |
