# Supplementary material for: Chemical, Target, and Bioactive Properties of Allosteric Modulation
Source: PLoS Comput Biol. 2014 Apr 3;10(4):e1003559. doi: 10.1371/journal.pcbi.1003559 (PMC3974644; doi:10.1371/journal.pcbi.1003559)
Supplement: Table S6 — Allosteric models for balanced data sets of L0, L1, and L2 groups. (DOCX) [file pcbi.1003559.s010.docx]

# Table S6: Allosteric models for balanced data sets of L0, L1, and L2 groups.

| Target Level | Class | Allosteric | Non-allosteric | Sens | Spec | PPV | NPV | Allosteric property 1 | Allosteric property 2 | Allosteric property 3 | Non-allosteric Property 1 | Non-allosteric Property 2 | Non-allosteric Property 3 |
| --- | --- | --- | --- | --- | --- | --- | --- | --- | --- | --- | --- | --- | --- |
| 0 | n/a | 18,281 | 18,035 | 0.83 | 0.82 | 0.82 | 0.83 | Doublebonds Frac | Carbon Frac | LogP | drugLikeness | PSA Fraca | StereoAtom Frac |
| 1 | Adhesion | 23 | 91 | 1.00 | 0.91 | 0.67 | 1.00 | Stereobonds Frac | Formal Charge | Sulphur Frac | Rotatable Bonds Frac | PSA Frac | Polar SASA Frac |
| 1 | Cytosolic Other | 259 | 279 | 0.88 | 0.89 | 0.88 | 0.89 | Solubility | Lipinski Pass | Sulphur Frac | Molecular SASA | Molecular Weight | Num Aromatic Rings |
| 1 | Enzyme | 9,531 | 8,425 | 0.84 | 0.81 | 0.83 | 0.82 | DoubleBonds Frac | Aromatic Bonds Frac | Sulphur Frac | drugLikeness | Molecular PSA | Num Chains |
| 1 | Ion Channel | 1,974 | 1,966 | 0.86 | 0.88 | 0.88 | 0.86 | LogD | LogP | Sulphur Frac | Solubility | H Donor Frac | Heteroatom Frac |
| 1 | Membrane Other | 0 | 752 | n/a | n/a | n/a | n/a | n/a | n/a | n/a | n/a | n/a | n/a |
| 1 | Membrane Receptor | 4,617 | 4,927 | 0.86 | 0.88 | 0.87 | 0.89 | Aromatic Bonds Frac | sp2 Carbon Frac | Ringbonds Frac | Nitrogen Frac | Stereoatom Frac | Positive Atom Frac |
| 1 | Nuclear Other | 0 | 284 | n/a | n/a | n/a | n/a | n/a | n/a | n/a | n/a | n/a | n/a |
| 1 | Secreted | 141 | 179 | 0.83 | 0.95 | 0.92 | 0.88 | Doublebonds Frac | drugLikeness | Cmp Acid | LogD | Cmp Neutral | Singlebonds Frac |
| 1 | Structural | 2 | 1,876 | n/a | n/a | n/a | n/a | n/a | n/a | n/a | n/a | n/a | n/a |
| 1 | Surface Antigen | 0 | 529 | n/a | n/a | n/a | n/a | n/a | n/a | n/a | n/a | n/a | n/a |
| 1 | TranscriptionFactor | 1,147 | 1,159 | 0.89 | 0.85 | 0.86 | 0.89 | Molecular SASA | Negative Atoms Frac | Cmp Acid | H Donors Frac | Aliphatic Ringbonds Frac | Nitrogen Frac |
| 1 | Transporter | 203 | 196 | 0.82 | 0.83 | 0.84 | 0.81 | Bridgebonds Frac | Molecular Weight | Molecular Surface Area | Aliphatic Ringbonds Frac | Cmp Organic Non-peptidic | drugLikeness |
| 1 | Undefined | 384 | 585 | 0.83 | 0.94 | 0.87 | 0.90 | LogP | Carbon Frac | LogD | Molecular Volume | StereoAtom Frac | Num sp2 Carbons |
| 2 | 7TM1 | 2,130 | 2,081 | 0.89 | 0.89 | 0.89 | 0.89 | Rigidity Index | Carbon Frac | Polar SASA Frac | Num sp3 Carbons | Hydrogen Frac | Sp3 Carbon Frac |
| 2 | 7TM2 | 119 | 107 | 1.00 | 0.88 | 0.90 | 1.00 | sp2 Carbon Frac | Carbon Frac | Rigidity Index | Num Chains | SP3 Carbon Frac | Singlebonds Frac |
| 2 | 7TM3 | 2,192 | 2,196 | 0.91 | 0.88 | 0.89 | 0.91 | Num Chain Assemblies | Carbon Frac | LogP | Positive Atom Frac | Sp3 Carbon Frac | Cmp Zwitterion |
| 2 | Aminoacyl-transferase | n/a | n/a | n/a | n/a | n/a | n/a | n/a | n/a | n/a | n/a | n/a | n/a |
| 2 | ASIC | n/a | n/a | n/a | n/a | n/a | n/a | n/a | n/a | n/a | n/a | n/a | n/a |
| 2 | Cytochrome P450 | 193 | 192 | 0.90 | 0.75 | 0.78 | 0.88 | Sulphur Frac | Cmp Species Undefined | Aliphatic Ringbonds Frac. | Rotatable Bonds Frac | Carbon Frac | Molecular SASA |
| 2 | Electro-chemical | 94 | 85 | 0.96 | 0.85 | 0.88 | 0.94 | Molecular Volume | Molecular Weigh | Num Chains | LogD | Solubility | LogP |
| 2 | IP3 | n/a | n/a | n/a | n/a | n/a | n/a | n/a | n/a | n/a | n/a | n/a | n/a |
| 2 | Kinase | 1,461 | 1,419 | 0.90 | 0.88 | 0.89 | 0.90 | Oxygen Frac | Molecular SASA | Num Terminal Rotomers | Nitrogen Frac | Ringbonds Frac | Num Rings |
| 2 | KIR | n/a | n/a | n/a | n/a | n/a | n/a | n/a | n/a | n/a | n/a | n/a | n/a |
| 2 | LGIC | 1,803 | 1,791 | 0.86 | 0.89 | 0.89 | 0.86 | Sulphur Frac | Num Chains | Doublebonds Frac | Solubility | H acceptor Frac | Positive Atom Frac |
| 2 | NTPase | 109 | 111 | 0.96 | 0.83 | 0.85 | 0.95 | H Donor Frac | Num sp2 Carbons | Sp2 Carbon Frac | Aliphatic Ringbonds Frac. | Num Aliphatic Rings | Num Chain Assemblies |
| 2 | Nuclear Receptor | 1,129 | 1,092 | 0.90 | 0.84 | 0.86 | 0.88 | Molecular Weight | Negative Atom Frac | Cmp Acid | H Donors Frac | Aromatic Bonds Frac | Aliphatic Ringbonds Frac. |
| 2 | Phosphatase | 92 | 81 | 0.86 | 0.84 | 0.86 | 0.84 | LogD | Aliphatic Ringbonds Frac | Num Aliphatic Rings | Polar SASA Frac | Negative Atom Frac | Rotatable Bonds Frac. |
| 2 | Phospho-diesterase | 43 | 40 | 0.75 | 0.71 | 0.75 | 0.71 | Negative Atom Frac | Oxygen Frac | Num sp2 Carbonsc | Positive Atom Frac | drugLikeness | Heteroatom Frac |
| 2 | Protease | 600 | 571 | 0.88 | 0.87 | 0.88 | 0.87 | Oxygen Frac | Doublebonds Frac | Carbon Frac | Nitrogen Frac | H Donor Frac | Num Ring Assemblies |
| 2 | Reductase | n/a | n/a | n/a | n/a | n/a | n/a | n/a | n/a | n/a | n/a | n/a | n/a |
| 2 | RYR | 11 | 14 | 0.50 | 0.67 | 0.50 | 0.67 | Aromatic Bonds Frac | Num Halogens | Num Ring Assemblies | Num Aliphatic Rings | H Acceptor Frac | Singlebonds Frac |
| 2 | SUR | 11 | 13 | 1.00 | 1.00 | 1.00 | 1.00 | Nitrogen Frac | Sulphur Frac | Solubility | Molecular Volume | Molecular Surface Area | Num sp2 Carbons |
| 2 | TRP | 43 | 39 | 1.00 | 1.00 | 1.00 | 1.00 | Molecular Surface Area | Molecular PSA | Rotatable Bonds Frac | Num Terminal Rotomers | Ringbonds Frac | Rigidity Index |
| 2 | Undefined | 8,145 | 8,094 | 0.84 | 0.85 | 0.85 | 0.84 | Aromatic Bonds Frac | Solubility | Suplhur Frac | drugLikeness | Num Chains | Molecular Polar Surface Area |
| 2 | VGC | 106 | 109 | 0.96 | 0.92 | 092 | 0.96 | Ringbonds Frac | Num Halogens | Polar SASA Frac | Num Chains | Hydrogen Frac | Molecular Volume |

Abbreviations: 7TM1 – Class A GPCRs, 7TM2 – Class B GPCRs, 7TM3 – Class C GPCRs, ASIC – Acid Sensing Ion Channels, IP3 – Inositol trisphosphate receptors, KIR – Killer-cell Immunoglobulin-like Receptors, LGIC – Ligand Gated Ion Channels, RYR – Ryanodine Receptors, SUR – Sulfonylurea Receptors, TRP – Transient receptor potential channels, VGC – Voltage Gated Ion Channels, Frac – Frac, Cmp – compound, H Acceptors – Hydrogen Bond Acceptors, H Donors – Hydrogen Bond Donors, LogD – distribution coefficient, LogP – partition coefficient, PSA – Polar Surface Area, SASA – Solvent Accessible Surface Area, sp2 – SP3 hybridized Carbons, sp3 – SP3 Hybridized Carbons, Num – Number of, n/a – Not Available.
