# Supplementary material for: Chemical, Target, and Bioactive Properties of Allosteric Modulation
Source: PLoS Comput Biol. 2014 Apr 3;10(4):e1003559. doi: 10.1371/journal.pcbi.1003559 (PMC3974644; doi:10.1371/journal.pcbi.1003559)
Supplement: Table S10 — Keywords used to retrieve the allosteric set. (DOCX) [file pcbi.1003559.s014.docx]

# Table S10: Keywords used to retrieve the allosteric set

| ﻿Keyword | Frequency in Abstracts  (documents) | Frequency in  ChEMBL-14  (documents) |
| --- | --- | --- |
| activators | 2532 | 266 |
| allosteric | 1811 | 274 |
| allosterism | 7 | 0 |
| allostery | 41 | 0 |
| alosteric | 0 | 0 |
| alosterism | 0 | 0 |
| alostery | 0 | 0 |
| indirectly activate | 16 | 0 |
| indirectly inhibit | 21 | 0 |
| negative modulator | 46 | 4 |
| negative modulators | 0 | 0 |
| NNRTI | 140 | 47 |
| non-competitive | 258 | 76 |
| non-nucleoside reverse transcriptase inhibitor | 56 | 11 |
| Noncompetitive | 399 | 150 |
| nonsubstrate | 37 | 5 |
| positive modulator | 26 | 3 |
| positive modulators | 0 | 0 |
| regulatory site | 171 | 1 |
| secondary binding site | 34 | 6 |
| secondary pocket | 15 | 14 |
| un-competitive | 0 | 0 |
| Uncompetitive | 109 | 35 |
| Multiple hits | 456 | 95 |
| **Total** | **6175** | **987** |
